# Syntrophic entanglements for propionate and acetate oxidation under thermophilic and high-ammonia conditions

**DOI:** 10.1038/s41396-023-01504-y

**Published:** 2023-09-07

**Authors:** Abhijeet Singh, Anna Schnürer, Jan Dolfing, Maria Westerholm

**Affiliations:** 1 Department of Molecular Sciences, Swedish University of Agricultural Sciences, SE-750 07 Uppsala, Sweden; 2 Faculty of Energy and Environment, Northumbria University, Newcastle-upon-Tyne, NE18QH UK

**Keywords:** Applied microbiology, Metagenomics, Metagenomics, Metagenomics

## Abstract

Propionate is a key intermediate in anaerobic digestion processes and often accumulates in association with perturbations, such as elevated levels of ammonia. Under such conditions, syntrophic ammonia-tolerant microorganisms play a key role in propionate degradation. Despite their importance, little is known about these syntrophic microorganisms and their cross-species interactions. Here, we present metagenomes and metatranscriptomic data for novel thermophilic and ammonia-tolerant syntrophic bacteria and the partner methanogens enriched in propionate-fed reactors. A metagenome for a novel bacterium for which we propose the provisional name ‘*Candidatus* Thermosyntrophopropionicum ammoniitolerans’ was recovered, together with mapping of its highly expressed methylmalonyl-CoA pathway for syntrophic propionate degradation. Acetate was degraded by a novel thermophilic syntrophic acetate-oxidising candidate bacterium. Electron removal associated with syntrophic propionate and acetate oxidation was mediated by the hydrogen/formate-utilising methanogens *Methanoculleus* sp. and *Methanothermobacter* sp., with the latter observed to be critical for efficient propionate degradation. Similar dependence on *Methanothermobacter* was not seen for acetate degradation. Expression-based analyses indicated use of both H_2_ and formate for electron transfer, including cross-species reciprocation with sulphuric compounds and microbial nanotube-mediated interspecies interactions. Batch cultivation demonstrated degradation rates of up to 0.16 g propionate L^−1^ day^−1^ at hydrogen partial pressure 4–30 Pa and available energy was around −20 mol^−1^ propionate. These observations outline the multiple syntrophic interactions required for propionate oxidation and represent a first step in increasing knowledge of acid accumulation in high-ammonia biogas production systems.

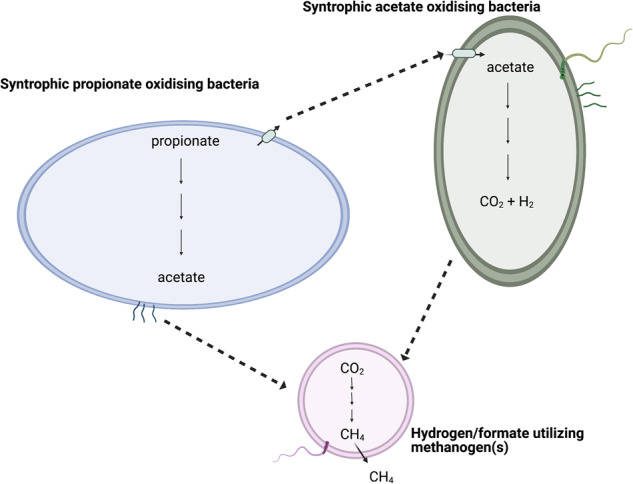

## Introduction

Through anaerobic digestion, waste is efficiently converted to a combustible gas, comprising methane (CH_4_) and carbon dioxide (CO_2_). This is one of the most sustainable options for renewable energy production when accounting for the additional benefits, such as abated greenhouse gas emissions, efficient waste management, recovery of nutrients and substitution of synthetic and mineral fertiliser when using the residue as bio-fertiliser [1]. The anaerobic degradation process involves a series of microbial degradation steps engaging different anaerobic microorganisms, often operating at near thermodynamic equilibrium [2, 3]. In the process, complex organic materials are initially hydrolysed to sugars, amino acids and long-chain fatty acids that are further fermented to volatile fatty acids (VFA), alcohols and smaller amounts of hydrogen (H_2_). In the following anaerobic oxidation step, VFA longer than acetate, such as propionate and butyrate, are degraded to acetate, H_2_ and CO_2_, which in a terminal step are converted to CH_4_ and CO_2_ by methanogenic archaea. Although the anaerobic digestion process is generally operated in continuous/semi-continuous mode, disturbances can occur during operation, triggered, for instance, by a change in feeding composition/rate, temperature fluctuations, trace element deficiency or ammonia toxicity [4]. A pervasive consequence, particularly with ammonia-induced perturbations if the anaerobic digester is fed protein-rich substrates, is accumulation of propionate and acetate, which exposes the process disturbance [5, 6]. At high ammonia levels, acetate accumulation is often caused by inactivation of acetate-cleaving methanogens, opening up an opportunity for development of an alternative acetate-degrading community involving a synergy between syntrophic acetate oxidisers (SAOB) and hydrogenotrophic methanogens (HM) [7]. The exact cause of propionate accumulation at high ammonia concentrations is poorly understood, but may relate to suppression of ammonia-sensitive propionate-degrading communities [6, 8, 9] and a temporal lapse in establishment of ammonia-tolerant propionate degraders [10].

Propionate is degraded through interactions between syntrophic propionate-oxidising bacteria (SPOB) and hydrogen- and/or formate-utilising HM. Acetate is subsequently degraded by aceticlastic methanogens in low ammonia conditions [11, 12] or by SAOB and hydrogen- and/or formate-utilising HM in high-ammonia conditions [13]. The SPOB characterised to date are from the families *Peptococcaceae* and *Syntrophobacteraceae*, which all degrade propionate using the methylmalonyl-CoA pathway or the dismutating pathway (only *Smithella*) [10]. SPOB candidates include members of the phylum *Cloacimonadota* found in both mesophilic and thermophilic biogas processes [14, 15], ‘*Candidatus* Propionivorax syntrophicum’ discovered in a mesophilic wastewater treatment plant [16] and the only known ammonia-tolerant SPOB, ‘*Candidatus* Syntrophopropionicum ammoniitolerans’ identified from a mesophilic biogas process [13]. The majority of these SPOB originate from biogas reactors, clearly demonstrating that syntrophic propionate oxidation (SPO) is a distinct feature of the anaerobic digestion process. However, although high-ammonia anaerobic digestion has been widely studied from a process perspective, there are indications that several acid-degrading microorganisms with key roles in that process have not been identified, isolated or characterised [10, 13]. Consequently, the reciprocal communal interactions within and between such communities, i.e. between SAOB, SPOB and HM, are currently underexplored. In particular, current understanding of SPO in thermophilic and high-ammonia biogas processes and the cross-species interactions enabling stepwise conversion of propionate to methane is limited.

In the present study, a thermophilic SPOB community in high-ammonia conditions was enriched and its molecular exchange and interaction network with SAOB and HM were analysed. The microbial communities were explored through both 16S rRNA gene amplicon sequencing and whole metagenome sequencing, and their physiological activity during propionate and acetate degradation was characterised using metatranscriptomics. Chemical monitoring of batch trials of propionate- and acetate-supplemented cultures was performed to study the effects of intermediate product formation and consumption on propionate- and acetate-degradation kinetics.

## Materials and methods

### Continuously fed reactor set-up and operation

Four identical laboratory-scale continuously stirred (80 rpm) tank reactors (Belach Bioteknik) with working volume 1.1 L were operated at 52 °C. Separate pairs of reactors were designated for propionate (RP1, RP2) and acetate (RA1, RA2) enrichment. The reactors were continuously fed with anoxic, sterile bicarbonate-buffered basal medium (BM) [17] supplemented with 8.9 g L^−1^ ammonium chloride (3 g L^−1^ NH_4_^+^-N) and 0.1 M (9.6 g L^−1^) sodium propionate (RP1, RP2) or 0.1 M (8.2 g L^−1^) sodium acetate (RA1, RA2). The initial pH of the BM was 7.3 and the reactors were inoculated with 1 L sterile BM and 0.1 L sludge from a thermophilic and high-ammonia large-scale biogas digester [18] (Table S1) under flushing with N_2_. The four reactors were fed with peristaltic pumps (Belach Bioteknik) at a dilution rate of 26 µL min^−1^, resulting in a hydraulic retention time of 28 days. Concentrations of short-chain VFA (acetate, propionate, butyrate, isobutyrate, valerate, isovalerate, capronate and isocapronate) were analysed using high performance liquid chromatography (HPLC), and methane content of the gas was determined by gas chromatography (GC) as described previously [19]. Volume of gas produced was measured continuously using µFlow (2 mL resolution, BioProcess Control). The VFA and gas production were monitored weekly for the first 320 days and thereafter occasionally to confirm the stable conditions till the end of the experiment at day 630.

### Batch experiment set-up

To evaluate the kinetics of the cultures enriched in the different reactors, anaerobic batch assays were prepared by transferring 0.5 L of the enrichment culture from the continuously propionate- and acetate-fed reactors after 630 days of operation directly to sterile anaerobic serum bottles (1 L) under constant N_2_ flushing. After a few days of acclimatisation at 52 °C without stirring, sodium propionate or sodium acetate was added to the batch culture, with the intention to reach a final concentration of 30 mM (due to variation in VFA levels in the culture transferred from the reactors, the initial acid concentration range after addition in the batch assays was 21–37 mM propionate and 34–80 mM acetate). Duplicate batch assays were prepared for each set-up and reactor, giving a total of 12 batch assays (B01-B12). Incubation was at 52 °C (pH 8.5 ± 0.1) at a free ammonia level of 1.2 g NH_3_ L^-1^. Cultures without addition of propionate or acetate served as negative controls. Determination of gas composition (H_2_, CH_4_, CO_2_) and analyses of liquid samples (acetate, propionate) were conducted. Hydrogen partial pressure was measured as described elsewhere [20]. The concentrations of acetate, propionate and formate after cessation of methane formation (after 50–86 days) were determined by nuclear magnetic resonance spectroscopy as described in Supplementary note 1.

### Thermodynamic calculations

For the thermodynamic calculations, the chemical reactions were evaluated using equations 1–3 (Supplementary note 2). Doubling time (t_d_) was estimated from specific methane production rate (µ_CH4/P/A_) as t_d,CH4/P/A_ = ln2/µ_CH4/P/A_, where µ_CH4/P/A_ was calculated from the slope of logarithmic methane, propionate or acetate change during exponential acid degradation and methane production.

1 $$\begin{array}{cc}{{{{{\rm{C}}}}}}{{{{{{\rm{H}}}}}}}_{3}{{{{{\rm{CO}}}}}}{{{{{{\rm{O}}}}}}}^{-}+{{{{{{\rm{H}}}}}}}^{+}+2{{{{{{\rm{H}}}}}}}_{2}{{{{{\rm{O}}}}}}\to 2{{{{{\rm{C}}}}}}{{{{{{\rm{O}}}}}}}_{2}+4{{{{{{\rm{H}}}}}}}_{2}{{{{{\rm{O}}}}}} & \Delta G^\circ =55.0{{{{{\rm{kJ}}}}}}\end{array}$$

2 $$\begin{array}{cc}4{{{{{{\rm{H}}}}}}}_{2}+{{{{{\rm{C}}}}}}{{{{{{\rm{O}}}}}}}_{2}\to {{{{{\rm{C}}}}}}{{{{{{\rm{H}}}}}}}_{4}+2{{{{{{\rm{H}}}}}}}_{2}{{{{{\rm{O}}}}}} & \Delta G^\circ =-130.8{{{{{\rm{kJ}}}}}}\end{array}$$

3 $$\begin{array}{cc}{{{{{\rm{C}}}}}}{{{{{{\rm{H}}}}}}}_{3}{{{{{\rm{CO}}}}}}{{{{{{\rm{O}}}}}}}^{-}+{{{{{{\rm{H}}}}}}}^{+}\to {{{{{\rm{C}}}}}}{{{{{{\rm{H}}}}}}}_{4}+{{{{{\rm{C}}}}}}{{{{{{\rm{O}}}}}}}_{2} & \Delta G^\circ =-75.8{{{{{\rm{kJ}}}}}}\end{array}$$

### DNA extraction, 16S rRNA gene amplicon sequencing, qPCR and data analysis

Total DNA was extracted from samples taken from reactors and batch assay on 19 and 8–10 occasions, respectively. The preparation of samples for MiSeq paired-end (2 × 300 bp) (Illumina) 16S rRNA gene amplicon sequencing is described in Supplementary note 3. Illumina adapters and primer sequences were trimmed using BBMap (v38.61b) [21]. Generation of amplicon sequence variants (ASV), abundance table and taxonomic assignment of ASVs were performed using the package *dada2* (v1.22.0) [22] in R (v4.1.3) [23]. Taxonomic classification of the ASVs was carried out using the GTDB taxonomic training dataset v202 formatted for DADA2 [24]. A phyloseq object was created using abundance and taxonomy tables and community structure was visualised using the package *phyloseq* (v1.38.0) [25] in RStudio (v2022.02.3 + 492) [26]. Quantitative PCR (qPCR) was performed to determine 16S rRNA gene copy number of methanogens in the orders *Methanomicrobiales* and *Methanobacteriales*, using primers and conditions as described previously [27, 28].

### Metagenomic sequencing, assembly, binning and functional analysis

Whole metagenome sequencing on samples from the parallel propionate-fed digesters (RP1 and RP2) withdrawn at day 115 was performed using HiSeq (Illumina), with 2 × 150 bp pair-end reads (Eurofins, Germany). For long read sequencing, genomic high molecular weight DNA extraction, sample preparation and sequencing using a MinION device (Oxford Nanopore Technologies) were performed as described in Supplementary note 3. Hybrid genome assembly was performed using flye (v2.8) [29, 30], racon (v1.4.13) [31] and medaka (v1.0.3) [32]. Refinement of subsequent long read assembly was done with short reads using multiple polishing rounds with Pilon (v1.23) [33]. Metagenomic binning was done using metaWRAP pipeline [34] and quality of resulting bins was accessed using CheckM (v1.0.18) [35]. Taxonomic classification of metagenome assembled genomes (MAGs) was performed with GTDB-tk (v1) [36], using the GTDB database (R202) [37]. Functional annotation of MAGs was done using Bakta (v1.4.1) [38] for bacterial and Prokka (v1.14.6) [39] for archaea (see Supplementary note 4 for details).

Annotated MAGs were manually validated for the genes involved in the methylmalonyl-CoA pathway and hydrogenase gene sequences extracted from the MAGs were manually classified against hydrogenase database HydDB (accessed June 2022) [40]. Digital DNA-DNA hybridisation (dDDH) between retrieved MAGs and genomes of closest relatives were determined using the GGDC (v3.0) [41]. Whole-genome average nucleotide identity (ANI) and average amino acid identity (AAI) were calculated using Pyani (blast+ algorithm) (v0.2.10) [42] and CompareM [43], respectively. Visualisation of ANI and AAI results was performed in RStudio with the package *ggplot2*. Species trees for the methanogens and SPOBs were created by the STAG method implemented in Orthofinder (v2.5.4) [44, 45]. Visualisation and annotation of all phylogenetic trees was performed with package *ggplot2* (v3.3.2) [46] and Figtree (v1.4.3) [47].

### RNA extraction and analysis of transcriptomics data

For RNA extraction, 50 mL volume of microbial culture were withdrawn from the batch assays (propionate B01 and B03, acetate B09) during the exponential phase of acetate and propionate degradation. Each culture sample was anaerobically transferred to a Falcon tube and immediately centrifuged at 5000 g and 4 °C for 10 min. The cell pellet was dissolved in 1 mL Trizol and 0.2 mL chloroform, and total RNA was extracted using the Quick-RNA Fecal/Soil Microbe Microprep Kit with an additional DNase I depletion step [48]. A two-step ribosomal RNA depletion protocol was employed using pan-prokaryotes riboPOOL probes and Dynabeads (MyOne Streptavidin C1, Invitrogen #65001) according to the manufacturer’s protocol [49]. Ribosomal RNA-depleted RNA samples were used for paired-end (2 × 75 bp) MiSeq sequencing (Illumina, v3 chemistry) on the SNP&SEQ platform [50]. The raw RNA sequence data were processed with BBMap (v38.61b) for quality control and removal of ribosomal RNA reads. Quantification (transcripts per million, TPM) of the filtered reads was performed by mapping against the MAGs using Salmon (v1.6.0) [51]. Quantification results were used for differential gene expression and other analyses in Rstudio, with the packages *DESeq2* (v1.37.0) [52], *ggplot2* and *pheatmap* (v1.0.12) [53]. All values used and represented in heatmaps are based on TPM counts (Supplementary data).

## Results and discussion

### Reactor performance revealed temporal changes in propionate degradation rate

The four propionate- and acetate-fed reactors used in the study produced biogas with an average methane content of 62–70% (Table S2). The pH was 8.1–8.3, resulting in an ammonia-nitrogen level of 0.7–0.9 g NH_3_ L^-1^. This free ammonia level is well above the threshold at which many microorganisms are inhibited, frequently causing reductions in overall methane production and accumulation of VFAs even in ammonia-adapted biogas processes [54].

In agreement with previous findings for thermophilic acetate-fed reactors [19], acetate content remained stable at around 0.7–0.9 g L^−1^ in the acetate-fed reactors RA1 and RA2. However, VFA degradation in the propionate-fed reactors was less stable. Propionate fluctuations (0.8–3.9 g L^−1^) were especially pronounced in reactor RP2 during the latter stages of operation (days 200–320, Fig. S1). As a result of changes in propionate level, acetate level in RP2 fluctuated from below detection to 3.5 g L^−1^ throughout the operating period. Other VFAs analysed were not detected above the detection limit of 0.2 g L^−1^ in the reactors. Comparing thermophilic reactor performance with that in previous mesophilic enrichment study [13], revealed less efficient propionate removal in thermophilic than in the mesophilic propionate reactors (Table S4). One reason for this may be the somewhat higher pH and associated higher ammonia levels in the thermophilic than in the mesophilic reactors.

### Batch assays and thermodynamics

For reasons unclear, it was not possible to initiate propionate-degrading activity in batch assays by preparing culture media and inoculating with 5–20% (v/v) of culture from the continuously fed reactors, a procedure routinely applied with success for mesophilic propionate-degrading cultures (unpublished data) originating from analogous mesophilic reactor experiment [13]. Propionate and acetate-degradation rates were therefore analysed by adding these VFAs to batches consisting of undiluted cultures from the continuously fed reactors. The degradation rates of propionate and acetate, H_2_ level and methane production rate were similar in the duplicate batches originating from the same reactor, but the degradation rate of propionate differed between the RP1 and RP2 communities (Fig. S2). The communities originating from RP1 (batches B01-B02) had the highest propionate-degradation rate and degraded the added propionate within 25 days. The rate of propionate degradation in the RP2 batches was considerably lower, and up to 100 days were required for complete propionate degradation. In batches B01-B02 propionate was consumed and methane formed according to the expected stoichiometry of 1.75 mol methane per mol propionate, whereas in batches B03-B04 the methane yield was slightly higher than expected (Table S3). Hydrogen levels were relatively similar in the propionate-fed batch cultures, foremost ranging between 3.5 and 12 Pa (Table S5), and were in line with levels previously reported for thermophilic acetate-oxidising cultures [55, 56], but somewhat higher than values reported for mesophilic propionate-oxidising communities [20, 57].

The underlying cause of the slower propionate degradation of the RP2 community compared with the RP1 community is unknown. However, the relatively similar acetate-degradation rates and H_2_ partial pressures in all communities (Table S5) indicate that the slower propionate degradation in RP2 was not because of low activity of the acetate-degrading community or slow removal of H_2_ in this reactor. Thus, the SPOB in RP2 were most likely inhibited by another factor. Furthermore, the relatively rapid propionate degradation in the RP1 batches (B01-B02) resulted in formation of 2–3 g acetate L^−1^ before the SAOB initiated acetate degradation (Fig. S2). The accumulated acetate and the calculated rate of propionate and acetate degradation in the present study also indicated that the SPOB community was able to degrade propionate at a rate that exceeded the acetate-degrading capacity of SAOB (Fig. S2, Table S5). These results support previous observation of a peak in acetate concentration due to rapid propionate degradation in thermophilic conditions [58]. Similar elevations in acetate levels following propionate degradation have been observed in high-ammonia mesophilic reactors after VFA pulsing and in dairy manure digesters [59, 60]. However, in several studies of mesophilic and low-ammonia processes with aceticlastic methanogens as the main acetate degrader, the acetate concentration has remained at low levels despite degradation of 1–4 g propionate L^−1^ [16, 61–63]. One reason for the disparity between high- and low-ammonia conditions is possibly that acetate above a certain concentration may be required before initiation of acetate degradation by SAOB. Alternatively, less efficient HM activity at higher ammonia levels [64] increases the H_2_ and/or formate levels, impeding the activity of SPOB and SAOB. This emphasises the need to support the activity of both syntrophic bacteria and HM in order to obtain a stable process [13, 57, 63, 65–67]. In the continuously fed reactors in the present study, the relatively constant flow of acetate formed from the propionate was manageable by the SAOB community, in maintaining acetate level at <2.5 g L^−1^ (Fig. S1). This is important, since acetate at >4.8 g L^−1^ (80 mM) has been shown to severely restrict propionate oxidation [68].

The ∆G values calculated from measured parameters from the batch experiment varied somewhat between the different species, but for the SPOB the ∆G value for conversion of propionate to acetate and H_2_ fluctuated around −20 mol^−1^ propionate. For acetate oxidation to CO_2_ and H_2_ by the SAOB, the ΔG value was −10 to −30 kJ mol^−1^ acetate. For HM, the ΔG value was similar to that of propionate in both series of batches, although during the later stages of propionate degradation of the batches with slow propionate degradation (B03-B04) the ΔG values for SPOB were more favourable than those for HM (Fig. S3A). In the acetate fed batches, ΔG was consistently more favourable for HM than for SAOB. This agrees well with results previously obtained for mesophilic SAOB cultures, where the HM also obtained more energy than the SAOB (−20 and −10 kJ mol^−1^, respectively) [20]. Due to the dual syntrophy in the propionate-fed systems, the outcome changed when energy distribution was evaluated per mole of propionate mineralised to CH_4_ (Table S6). HM then gained most of the energy, as 1.75 moles of methane were generated for each mole of propionate mineralised. The dual syntrophy underpinning propionate degradation in these systems makes H_2_ one of the central intermediates, as it can be produced by both SAOB and SPOB, and low H_2_ levels are beneficial for both. For acetate, the interdependency is more complicated, i.e. the SPOB benefit from low levels, while the SAOB benefit from high levels. In the batch experiment, the average hydrogen levels were slightly lower in the propionate-fed batches than in the acetate-fed batches, irrespective of the acetate level (Figs. S3B, C). To gain further thermodynamic insights into the microbe interplay in the dual syntrophy and to unravel why and when the hydrogen scavenger operates at lower hydrogen levels in syntrophy with SPOB than with SAOB, future studies should monitor growth of the syntrophs and the methanogen under a set of constant H_2_ levels.

### Microbial community structure (16S rRNA gene) in enrichment reactors and batch assays

**Fig. 1:**
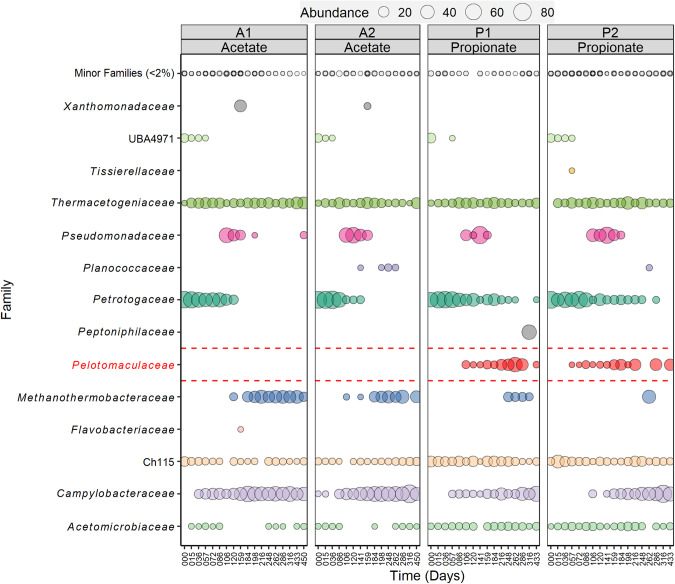
Microbial community structure resolving the exclusive presence of *Pelotomaculaceae* in the propionate-fed continuous reactors. Bubble plot showing percentage relative abundance (>2%) of microbial communities at family level in the acetate-fed (RA1, RA2) and propionate-fed (RP1, RP2) enrichment reactors.

Microbial community structure based on 16S rRNA gene amplicon sequencing most strikingly revealed enrichment of the family *Pelotomaculaceae* only in the propionate-fed reactors (RP1, RP2; 2–48%) and not in the acetate-fed reactors (RA1, RA2) (Fig. 1). *Pelotomaculaceae* harbours many known [10] and proposed SPOB [13, 16]. Major families observed throughout the experimental period in both the propionate- and acetate-fed reactors were *Acetomicrobiaceae* (3–13%), *Campylobacteraceae* (20–50%), Ch115 (5–20%) and *Thermacetogeniaceae* (2–34%). (Fig. 1). These five families were equally dominant in all batch assays except for family *Pelotomaculaceae*, which was specifically higher in relative abundance in the assays inoculated from the propionate-fed continuous reactors (B01-B08, Fig. S4). The batch assays from the propionate-fed reactors prepared with acetate as growth substrate (B05-B08) showed declining relative abundance (<5%) of *Pelotomaculaceae*, whereas its relative abundance was higher (3–50%) in batches fed propionate (B01-B04, Fig. S4). The enrichment of *Pelotomaculaceae* in propionate-fed continuous reactors (RP1, RP2) and its consistent presence at high relative abundances in propionate-based batch assays (B01-B04) indicate that members of this family were involved in SPO under the high-ammonia thermophilic conditions in the present study. The 16S rRNA gene sequencing indicated presence of two methanogenic species belonging to the genera *Methanoculleus* and *Methanothermobacter* in both acetate- and propionate-fed communities (Fig. 1, S4). These methanogenic genera have previously been suggested to be partners of a thermophilic *Pelotomaculum* sp. growing in low-ammonia conditions [69]. The absence of aceticlastic methanogens in the propionate-degrading community in the present study demonstrates the importance of the SAOB for acetate removal in high-ammonia conditions. Further detailed information of the sequencing result is given in Supplementary note 5.

### Metagenomic binning and metatranscriptomics-based functional analysis

Nine good-quality MAGs were obtained and based on taxonomic annotation and genomic content indicating putative involvement in syntrophic interactions, four of these MAGs were chosen for detailed analyses (Table S7). For instance, the MAGs affiliating to *Campylobacteraceae* and Ch115, highly abundant in the enrichment cultures, were shown to lack several of the crucial genes required for SAO/SPO-activity and were not included in further analyses. The four MAGs of interest for syntrophic acid degradation and their functional activities and their pathways are described in detail below.

### Novel SPOB of family *Pelotomaculaceae* ‘*Candidatus* Thermosyntrophopropionicum ammoniitolerans’

A high-quality MAG (MAG4, Table S7) classified to the family *Pelotomaculaceae* [phylum *Bacillota*_B, class *Desulfotomaculia*, order *Desulfotomaculales*] was recovered in metagenomic sequencing of samples from the propionate-fed reactors. No MAG with similar classification was recovered from the acetate-fed reactors. In a phylogenetic assessment based on whole-genome sequencing, MAG4 clustered together with ‘*Ca*. Propionivorax syntrophicum’, which further sub-clustered under SPOBs in the family *Pelotomaculaceae* (Fig. S5). Moreover, in an assessment based on 16S rRNA gene retrieval from MAGs, MAG4 showed a relationship to *Pelotomaculum* spp. and ‘*Ca*. Syntrophopropionicum ammoniitolerans’ (Fig. S6).

Comparison of MAG4 with available genomes of *Pelotomaculaceae* spp. and other known or proposed SPOBs revealed similarities below recommended cut-offs for delineating a new species (i.e. 70% dDDH, 95% ANI, 60% coverage) [70, 71]. MAG4 had highest similarities with ‘*Ca*. Propionivorax syntrophicum’ (dDDH of 42%, ANI of 90% and AAI of 89%). However, the genome assembly of ‘*Ca*. Propionivorax syntrophicum’ is of low quality (74.7% completeness), lack the 16S rRNA gene sequence and is considerably smaller (2.0 Mbp) than MAG4 (3.2 Mbp), which obstruct a complete and accurate comparison. The taxonomic analysis against other SPOB revealed highest dDDH with *Desulfofundulus thermobenzoicus* (24%), AAI with *P. thermopropionicum* (71%) and ANI with *Pelotomaculum schinkii* and *Pelotomaculum thermopropionicum* (74%) (<45% coverage) (Fig. S7). These results indicate that this bacterium will form a novel genus when isolated and characterised and we propose the provisional name ‘*Candidatus* Thermosyntrophopropionicum ammoniitolerans’.

### Methylmalonyl-CoA pathway

**Fig. 2:**
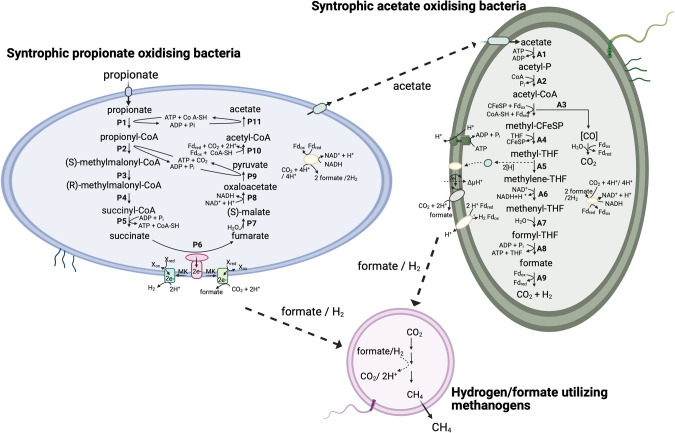
Metabolic reconstruction of syntrophic propionate and acetate oxidation and the interspecies hydrogen/formate transfer with hydrogenotrophic methanogens employed under thermophilic and high ammonia conditions. Visualisation of the molecular exchange anchored interplay and metabolic pathways employed by the multiple syntrophic bacteria and their methanogenic partner during syntrophic propionate degradation under thermophilic and high-ammonia conditions. The figure highlighted the cooperation of syntrophic propionate oxidising bacteria (SPOB, **MAG4**) via acetate assimilation by syntrophic acetate oxidising bacteria (SAOB, **MAG9**). These SPOB and SAOB further obligately establish formate or hydrogen pivoted syntrophic network to circumvent the reducing potential which is used by hydrogenotrophic methanogen (**MAG1**) to reduce carbon dioxide and generate methane.

**Fig. 3:**
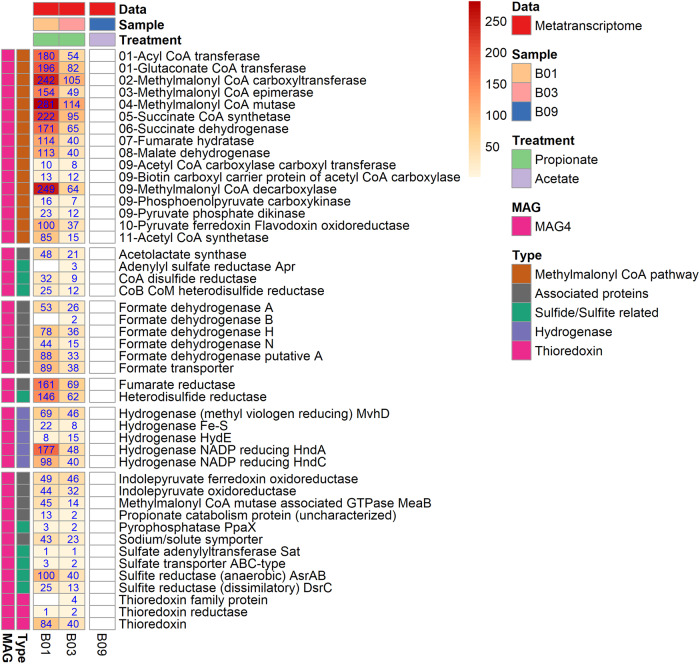
Gene expression profile of the methylmalonyl CoA pathway for propionate oxidation by the SPOB candidate. Metatranscriptomics expression profile of the methylmalonyl CoA (MMC) pathway of propionate metabolism (based on transcripts per million (TPM) counts) in propionate versus acetate batch assay (B01, B03 and B09) for the novel syntrophic propionate-oxidising bacteria (SPOB) `*Candidatus* Thermosyntrophopropionicum ammoniitolerans´ (**MAG4**). The numerical values with the enzyme name denote the step in the MMC pathway. The values on heatmap represented are the aggregated TPM counts of all copies and subunits for respective gene present and expressed in the metagenome assembled genome.

MAG4 harboured and expressed a complete set of genes required for propionate degradation through the methylmalonyl-CoA (MMC) pathway (Figs. 2, 3, S8), which strongly suggest that this bacterium can perform syntrophic propionate oxidation in high-ammonia, thermophilic biogas systems. MAG4 expressed CoA-transferase and carboxyltransferase. This indicates that, as in the thermophilic and mesophilic SPOB *P. thermopropionicum* and *P. schinkii* [72, 73], MAG4 coupled the two first endergonic steps, propionate activation (step P1 in Fig. 2) and propionyl-CoA carboxylation (P2), with the downstream and exergonic steps forming acetate (P11) and pyruvate (P9), respectively. Other genes encoding enzymes involved in the MMC pathway expressed by MAG4 include methylmalonyl-CoA epimerase, methylmalonyl-CoA mutase and succinate-CoA synthetase (P3-P5). To catalyse the energetically most unfavourable step in the MMC pathway, the oxidation of succinate to fumarate (P6), MAG4 expressed a gene encoding the membrane-bound succinate dehydrogenase/fumarate reductase complex, requiring reducing power via reverse electron transport. Genes encoding fumarate hydratase catalyses the conversion of fumarate to malate (P7) and malate dehydrogenase catalyses the conversion of malate into oxaloacetate (P8) were expressed. MAG4 also contained one Fe-S-containing hydrolyase which was annotated as fumarase/fumarate hydratase in *P. thermopropionicum* (BAF59538.1). The gene encoding pyruvate carboxylase in step P9 (Fig. 2), i*.*e. conversion of oxaloacetate to pyruvate, was not found in MAG4. However, the gene encoding methylmalonyl-CoA carboxyltransferase for step P2 (Figs. 2, 3) was expressed and this enzyme has been found to catalyse the conversion of oxaloacetate into pyruvate (KEGG reaction: R00930). For conversion of pyruvate to acetyl-CoA (P10), MAG4 encoded pyruvate:ferredoxin flavodoxin oxidoreductase.

In MAG4, most of the genes coding for the MMC pathway enzymes were found to be clustered together (except the genes for steps P1 and P6). The gene for propionate-CoA transferase (PCT) was not found in MAG4 (and is also absent in *P. thermopropionicum*). Instead, MAG4 expressed genes for two other CoA-transferases (acyl/acetate transferase, glutaconate-CoA transferase) that are homologous to PCT and have also been suggested to activate propionate [72, 74, 75]. These CoA-transferases genes were clustered in an operon fashion and highly expressed compared with other flanking genes in MAG4. MAG4 also expressed MMC pathway-associated genes, *viz*. formate and sodium/solute (propionate) transporters, formate dehydrogenase, methylmalonyl-CoA decarboxylase *etc*. (Figs. 2, 3). A gene encoding an uncharacterised protein likely involved in propionate catabolism (32% similarity to an uncharacterised protein (BAF60599.1) found in the *P. thermopropionicum* genome (AP009389.1)) was expressed by MAG4 (Fig. 3). This gene showed 100% similarity and query coverage (Blastx) to MmgE/PrpD family protein (NLW37044.1) belonging to a *Peptococcaceae* bacterium MAG (JAAYEO000000000.1). The PrpD family is involved in propionate oxidation to pyruvate in *E. coli* [76], but this protein has still not been annotated or characterised in other anaerobic bacteria. The expression of this gene by MAG4 indicate that this protein might be involved in propionate degradation by an as yet unknown mechanism.

### Hydrogen/formate production and energy conservation systems

In SPO, hydrogenases or formate dehydrogenases catalyse electron transfer from NADH or reduced ferredoxin (Fd, generated from substrate oxidation) to the final electron acceptors H^+^ and CO_2_ [12, 77]. MAG4 expressed genes for both hydrogenases and formate dehydrogenases (Fig. 3). More specifically, expression of genes for cytoplasmic [FeFe] electron-bifurcating- (HndAC) ([FeFe] group A3), membrane [Fe]-bound [NiFe] and iron-only hydrogenases ([FeFe] group A4) was revealed (Fig. S9). The latter has been shown to couple the endergonic formation of H_2_ from NADH to its exergonic formation from Fd_red_ [78]. In MAG4, the gene encoding Hnd was found to be flanked by expressed genes for formate dehydrogenase (FDH) and a formate transporter (FdhC) (Fig. 3, S8). It has been speculated that two FDH are needed for syntrophic growth on propionate, one for fixing CO_2_ by the reductive Wood-Ljungdahl pathway (the membrane-bound FDH1) and one for removal of reducing equivalents as format (the cytoplasmic FDH2) [79, 80]. For *P. thermopropionicum*, four types of FDH have been found [73] and these can be differentially and independently up- or down-regulated [81]. MAG4 expressed four FDH types (Fig. 3, S8), indicating the possibility that in MAG4, FDH could be utilising reducing equivalents (together with electron transfer/bifurcating flavoproteins and electron transport Rnf complex) to form H_2_ [82]. Further, formate transporter (transmembrane FocA) could be assisting in mediation of H_2_/formate-dependent electron sharing or electron bifurcation [83] between MAG4 (as SPOB) (Fig. 3, S9) and the HM, as also reported previously [16].

### Genome and transcriptomic analysis of candidate SAOB

For SAO, a distinct candidate (MAG9) was identified by genomic and transcriptomic analysis in both propionate- and acetate-degrading reactor communities. MAG9 was classified to genus DTU068 (95% dDDH similarity with place holder species sp001513545 in the phylum *Firmicutes*, class *Moorellia*, family *Thermacetogeniaceae*, Fig. S10). The taxonomic placement of MAG9 and the transcriptomic data in the propionate- and acetate-fed batch assays strongly indicate that this species represents a novel thermophilic SAOB, related to the mesophilic SAOB *Syntrophaceticus schinkii* (Figs. S11, S12). However, the genome sequence of MAG9 had high contamination (~15%) thus MAG quality was not sufficiently high for proposal of a provisional name for this species.

**Fig. 4:**
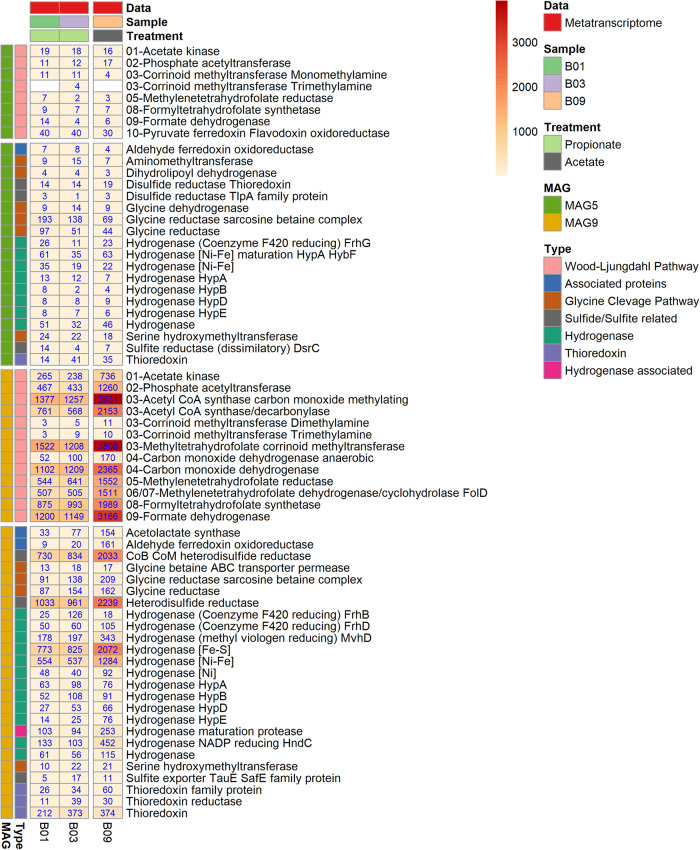
Gene expression profile of the Wood-Ljungdahl pathway by the SAOB candidate. Metatranscriptomics expression profile of the Wood-Ljungdahl pathway and other genes of relevance for acetate metabolism (based on transcripts per million (TPM) counts) in propionate versus acetate batch assay (B01, B03 and B09) for the novel syntrophic acetate-oxidising bacteria (SAOB) MAG9 and for MAG5 belonging to the genus *Acetomicrobium*. The numerical values with the enzyme name denote the step in the pathway. The values on heatmap represented are the aggregated TPM counts of all copies and subunits for respective gene present and expressed in the metagenome assembled genome.

MAG9 harboured and expressed a complete set of genes for the Wood-Ljungdahl pathway (WLP) in both propionate and acetate cultures (Fig. 4). The genome revealed a cluster of several WLP genes (steps A3-A6, A12 and A9 in Fig. 2) but for acetate activation (A1-A2) the genes were located separately and the transcriptome data indicated that MAG9 activates acetate through the ATP-consuming acetate kinase. Acetate can potentially also be activated through an ATP-independent aldehyde ferredoxin oxidoreductase followed by oxidation of acetaldehyde to acetyl-CoA, as postulated to be used by *Thermacetogenium phaeum* to balance the overall ATP budget [84]. Even though MAG9 encoded aldehyde ferredoxin oxidoreductase, the low transcript level of the encoding gene compared with the gene for acetate kinase indicates that, as seen in *S. schinkii* [85] MAG9 consumes ATP in this first step and forms acetyl-CoA using phosphate acetyltransferase. For the carbonyl branch, CO-methylating acetyl-CoA synthetase and carbon monoxide dehydrogenase were expressed (A4, Figs. 2, 4), whereas expression of corrinoid methyltransferases (A3), methylene tetrahydrofolate (THF) reductase (A5), methylene THF dehydrogenase/cyclohydrolase (A6, A7), formyl THF synthetase (A8) and format dehydrogenase (A9) indicated their importance in operation of the methyl branch (Figs. 2, 4).

In the direction of acetate oxidation, the methylene THF reductase (A5) releases electrons at a redox potential too low to be used directly for NAD^+^ reduction [86]. For *T. phaeum*, this has been proposed to be solved by electron transfer to a methyl-viologen-reducing hydrogenase subunit D (MvrD), followed by heterodisulphide reductase (HdrABC) and further to a quinone, which in turn is re-oxidised by formate dehydrogenase [84]. The gene expression seen for MvhD, HdrABC and NAD-quinone oxidoreductase, the four-iron-four-sulphur (4Fe-4S) cluster and 4Fe-4S ferredoxin by MAG9 indicate that a similar path is followed by this ammonia-tolerant SAOB (Fig. S12). Furthermore, expression of hydrogenase Fe-S, which was found to be encoded next to the genes for step A6 (Figs. 2, 4), indicates importance of electron transport and proton translocation. However, since the pathway proposed for *T. phaeum* requires establishment of a proton gradient from ATP hydrolysis (reverse electron transport), MAG9 metabolism would not generate enough ATP to drive acetate activation. Thus, further research is needed to confirm the metabolic route used by this and other SAOB. Similarly to *S. schinkii* and *T. phaeum*, [84, 85] MAG9 expressed hydrogenase EchCE, formate dehydrogenase and Ni-Fe hydrogenases in both acetate- and propionate-fed batches. MAG9 also expressed a complete set of genes for NADH-quinone oxidoreductase (Fig. S12).

### Additional active bacterium in the syntrophic consortia

A MAG (MAG5) belonging to the genus *Acetomicrobium* (76% [sourmash] and with 84% [dDDH] similarity to GCA_012518015.1) was present and showed activity in both acetate- and propionate-degrading cultures. This species expressed genes encoding the reductive glycine pathway (rGlyP), including the glycine cleavage system, the glycine reductase complex, pyruvate synthase and associated proteins (Fig. 4, S13). A detailed discussion regarding the role of MAG5 is given in Supplementary note 6, with the conclusion that considering the wide range of substrates used by members of *Acetomicrobium* [87–89] and that continuing cultivation of the syntrophic community in the present study demonstrated decreased abundance of *Acetomicrobium* on omitting yeast extract in the growth media (data not shown), MAG5 most likely fermented compounds included in the yeast extract or grew oxidatively using cysteine as electron acceptor.

### Genome and transcriptomics analysis of methanogen

The 16S rRNA gene sequencing analysis of the reactor microbial communities demonstrated higher abundance of *Methanothermobacter*_NA than *Methanoculleus* spp. (Fig. S14). The qPCR analyses demonstrated that *Methanomicrobiales* sp. decreased from 10^7-8^ to 10^5^ gene copies L^−1^ over the course of operation of the continuously fed reactors. Species belonging to *Methanobacteriales* were present at relatively stable levels over time, varying between 10^5^ and 10^7^ gene copies L^−1^ in all reactors (Table S8). However, in the batch assays, 16S rRNA gene amplicon sequencing analysis showed that *Methanothermobacter*_NA and ‘*Ca*. Methanoculleus thermohydrogenotrophicum’ were often present in similar relative abundance in batches from RP1. In batches from RP2, ‘*Ca*. Methanoculleus thermohydrogenotrophicum’ was the only dominant species (Fig. S15). This is in agreement with the qPCR results demonstrating higher abundance of *Methanobacteriales* in batches with faster propionate degradation (B01-B02, 10^6^ gene copies L^-1^) than in batches with slower propionate degradation (B03-B04, 10^4^ gene copies L^−1^) (Table S8). *Methanomicrobiales* sp. were present at 10^6^ gene copies L^−1^ in all batch assays. Taxonomic profiling using the custom krakan2 [90] database (Supplementary note 4) with the metagenomics data revealed that ‘*Ca*. Methanoculleus thermohydrogenotrophicum’ was the most dominant methanogen and that *Methanothermobacter* sp. was higher in relative abundance in reactor RP1 compared to RP2 (Fig. S16).

Metatranscriptomics results were in agreement with the 16S rRNA gene sequencing results from batch assays (Fig. S15), showing a high number of 16S rRNA transcripts mapped to *Methanoculleus* and *Methanothermobacter*, with a particularly high number of reads mapping to *Methanothermobacter* (default kraken2 database) (Fig. S17, Table S6) in the B01 having higher propionate degradation rate. Although *Methanothermobacter* was among the dominant methanogenic genera in the 16S rRNA gene sequencing, metagenomics and metatranscriptomics analyses (Figs. S14–S17), we were unfortunately not able to recover a MAG belonging to the genus *Methanothermobacter*, making it difficult to reveal the activity of this methanogen in the syntrophic cultures. However, a high-quality MAG (MAG1) was recovered and it showed relationship to ‘*Ca*. Methanoculleus thermohydrogenotrophicum’ (78% dDDH) (GCA_001512375.1) (Figs. S18, S19, Table S6). As reported for the ammonia-tolerant mesophilic *Methanoculleus bourgensis* [91], methanogenesis pathway genes were found to be clustered together in MAG1 (Fig. S20). Metatranscriptomic data revealed expression of genes by MAG1 coding the transferases, reductases and dehydrogenases needed for hydrogenotrophic metabolism, including methyl CoM-reductase and formylmethanofuran dehydrogenase (Fig. S20). Moreover, MAG1 expressed genes for the V-type ATP synthetase and alcohol dehydrogenases (Fig. S21). The latter has been found previously in *Methanoculleus* genomes, indicating a trait of using alcohol as electron donor [92]. As in other thermophilic *Methanoculleus* [69], the electron-bifurcating hydrogenases (coenzyme F_420_) (FrhABDG) and formate dehydrogenases (FdhABD) were expressed by MAG1 (Fig. S21). MAG1 also expressed genes for *hyp* type (HypABCDE), which encode proteins for expression and maturation of hydrogenases (Fig. S21). For the metabolic process and biosynthesis, MAG1 encoded and expressed acetate/acyl-CoA ligase (Fig. S21), which uses ATP for activation of acetate to acetyl-CoA. Metatranscriptomics quantification indicated almost identical expression pattern in MAG1 in both the propionate- and acetate-fed batch experiments. Overall, expression of the methanogenesis pathway and associated genes in MAG1 did not appear to give any specific differences in propionate versus acetate treatments that could reveal its partnership with SPOB or SAOB (Figs. S20, S21).

### Expression of other genes potentially related to a syntrophic lifestyle

Low energy gain is a well-known challenge and bottleneck for the thermodynamically constrained syntrophic interactions in microbial communities. Hence, it is reasonable to believe that strategies to reduce energy investment in cell metabolism is important for the species involved. To shed light on how the thermophilic ammonia-tolerant syntrophic communities in this study acclimatised to energy scarcity, particular attention was paid to activities with potential to increase energy gain and facilitate interspecies interactions.

For energy production, the candidate syntrophs, MAG4 and MAG9, both expressed the F_0_F_1_-type ATP synthase complex (Figs. S9, S12), as reported for known SAOB (*S. schinkii* and *T. phaeum*) [84, 85] and SPOB members of *Peptococcaceae* and *Syntrophobacteraceae* [10]. Both MAG4 and MAG9 express ATP synthase subunit C to a higher extent than the other subunits. Subunit C has been shown to be crucial for ion translocation that leverages the proton/sodium motive force across the cell membrane and prevents ion leakage [93, 94]. It has also been reported that the number of protons translocated is proportional to the number of subunit C [95] and that regulation of ATP synthase operon is proportional to ATP generation [96–98]. This suggests that this type of proton translocation mechanism could also be involved in the bioenergetics of SAO and SPO communities and efficient energy conserving ATP synthesis near thermodynamic equilibrium [97, 98]. Moreover, it has been suggested for the SPOB *P. thermopropionicum*, *P. schinkii* and *S. fumaroxidans* that presence of a reverse electron transfer mechanism, menaquinone loop and higher number of expressed genes encoding hydrogenases and formate dehydrogenase (and associated higher enzymatic and cellular activity) could provide more metabolic agility and flexibility in the case of varying hydrogen or formate consumption by the syntrophic methanogenic partner [72, 98, 99]. In MAG4 and MAG9, these genes are encoded as either more than one copy in the genome, or were highly expressed (or both), which further indicates that the proteins encoded by these genes play a critical role in the complex syntrophic interactions among acetate-/propionate-degrading and methanogenic communities at the thermodynamic borderline (Figs. 3, 4, S9, S12, Table S6).

For initiation of syntrophic oxidation, the SPOB and SAOB need to transport the substrate across the cell membrane, which can be done actively using a transport system or through passive diffusion [10]. The candidate SPOB MAG4 expressed sodium/solute transporter and both MAG4 and the candidate SAOB MAG9 expressed MFS transporter proteins for putative propionate intake (Figs. S9, S12). MFS transporters are broad-spectrum transport systems involved in uniport, symport or antiport of various cellular metabolites, sugars and organic acids [100] and have been reported to play a role in tolerance to high levels of acetate and propionate, for example in *Acetobacter* spp., *E. coli* and *P. putida* [101, 102]. Moreover, a gene belonging to the oxalate/formate antiporter (OFA) family of MFS transporters was located in the operon together with CoA-transferases and showed higher expression by MAG4 in the culture with faster propionate oxidisation relative to the culture with slower propionate degradation (B01 vs. B03, Fig. S9). This suggests that MFS transporters maybe responsible for acetate/propionate or formate transport in MAG4.

### The role of sulphur compounds in metabolic cooperation

The transcriptome data revealed activity related to sulphur metabolism by the candidate SPOB (Fig. S9), including *Hdr* and CoA-disulphide reductase (*cdr*). The *Hdr* gene complex has previously been found to assist in electron confurcation in *S. fumaroxidans* [99]. Moreover, MAG4 expressed genes for dissimilatory sulphite reductase (*dsrC*) and anaerobic sulphite reductase (*asrAB*), which are the key determinants for sulphur reduction-based energy conservation in sulphate-reducing bacteria [99, 103]. In MAG4, these sulphite reductase genes are encoded next to expressed putative NADH:ubiquinone oxidoreductase (*nfC*) and a *mvhD*, which are likely involved in electron transport phosphorylation or hydrogenase activities. Other important genes involved in dissimilatory sulphate reduction (adenylylsulphate reductase (*aprAB*), sulphate adenylyltransferase (*sat*), pyrophosphatase (*ppaX*), ABC-type sulphate transporter) are also encoded by MAG4, but were not highly expressed under the conditions investigated here. However, presence of all the genes required for sulphate reduction in MAG4 strongly indicates that this bacterium has the ability to respire sulphate if available. The ability of MAG4 to perform sulphate reduction and the exact mechanisms involved warrant further investigation, since this would improve understanding of another thermophilic SPOB, *P. thermopropionicum*. It is suggested to be a sulphate/thiosulphate/sulphite reducer [104], even though it has been described as unable to utilise sulphate due to absence of *aprB* and *dsrAB* genes [105, 106].

Similar sulphur metabolism potential as observed for MAG4 was observed for ‘*Ca*. Propionivorax syntrophicum’, possibly as a step in a series of reactions for sulphate-reducing metabolism, and a complex of *hdr*, *rnfC* and *dsr*, which could provide reduced ferredoxin for H_2_/formate production and also for low-energy metabolism [16, 99]. However, the cultivation medium in the present study contained no sulphate or sulphite, contradicting the suggestion that the enzymes are involved in metabolism of these sulphur compounds. Instead, the cultivation medium included Na_2_S, cysteine, yeast extract and sulphur-containing vitamins, i.e. biotin, thioctic acid and thiamine, which might give rise to hydrogen sulphide and other sulphur compounds [107, 108]. Many bacteria can also produce different di- and trisulphides [109, 110]. Furthermore, as discussed above, MAG5 was taxonomically related to species that can reduce cysteine to sulphide, indicating similar activity by MAG5 in the enrichment culture. MAG4 also expressed cysteine desulfurase (*icsS*), which is responsible for sulphur activation in the cysteine degradation pathway. However, it might also be involved in formation of amino acids, as observed for the SAOB *Schnuerera ultunensis*, in which this gene is associated with production of alanine and sulphane/persulphide sulphur intermediates from cysteine degradation [111–113]. Apart from cysteine serving as a reducing agent in the medium, it may also mediate the electron carrier in SPO and subsequent methanogenesis [114]. Protein trisulphides are of interest in the present context since they are involved in sulphur reduction machinery-based energy conservation. The DsrC associated protein trisulphide (metacycM:DsrC-trisulphides) can act as a key intermediate in the reversible redox reaction producing and consuming sulphite [103, 104]. Expression of *asrAB* and *dsrC* is a key determinant for sulphur reduction-based energy conservation in sulphate-reducing organisms [103]. Considering that several sulphate-reducing organisms have been found to establish syntrophic interactions in environments where sulphur is absent or limited [115], sulphate-reducing metabolic potential of MAG4 is further indicated. The genes for the sulphate-reducing pathway, together with different hydrogenases, could possibly also be involved in low-energy metabolism rather than sulphate reduction [99].

### Mobility and other features with potential to facilitate interspecies cooperation

MAG4 and MAG9 contained six and 23 motility associated proteins, respectively (Figs. S22, S23). One of the pilus-associated proteins in MAG4 is PilT (type IV pili) (Figs. S22, S23), which has been found to be associated with twitching motility, cellular adhesion, pilus retraction and sequence-specific DNA uptake [116, 117]. Further, MAG4, MAG5 and MAG9 all expressed TIGR00282 family metallophosphoesterase proteins which were similar to YmdB, characterised for its role in nanotube and biofilm formation and intercellular molecular exchange in *Bacillus subtilis* [118–121]. Different types of flagellar and pilus-related proteins are known to play a role in initiating cellular contact, biofilm formation and establishing syntrophy. For instance, *P. thermopropionicum* FliD is used to establish contact with the partner methanogen *M. thermautotrophicus* and to synchronise their metabolism [122, 123]. Considering the absence of the FliD gene in MAG4, it is likely that if this candidate SPOB uses direct interspecies electron transfer (DIET), it is employing a mechanism somewhat different from those characterised previously. Further, MAG4 and MAG9 expressed genes for cysteine synthase/O-acetylserine sulfhydrolase (CysK) and stage 0 sporulation protein (Spo0A) (Fig. S23), which can be involved in biofilm formation, as suggested for *Vibrio fischeri* (CysK) [124] and *B. subtilis* and *Clostridium difficile* (Spo0A) [125, 126]. This perhaps explains the absence of motility-related (flagella) proteins and strongly suggests that MAG4 and MAG9 use pilus appendages for physically establishing deep physical contact with each other and the syntrophic methanogenic partner when present in close proximity (PilT-mediated) or for biofilm formation or nanotube communication and intercellular molecular exchange [118, 127, 128]. This feature would resemble that in *P. thermopropionicum*, which is characterised for biofilm and nanowire formation and interspecies electron and hydrogen sharing when growing in syntrophy with *Methanothermobacter* [123, 129, 130]. Cross-cellular communication, signalling and quorum sensing is another important concept intrinsic to syntrophic associations. Quorum sensing and signalling mechanism-related genes were expressed in all MAGs (Fig. S23). These genes, together with other associated genes, e.g. for motility, signalling, biofilm formation have been shown to be involved in DIET, intercellular metabolite exchange and communications [131]. The exact mechanism of the cooperation (e.g. establishment of nanotubes, use of flagellar or pilar assemblies) used by the syntrophic bacteria and the methanogens warrants further investigation.

### Stress response

Expression of genes related to stress response, *viz*. chaperones (DnaJ, DnaK, ClpB, different chaperonins), heat shock protein (Hsp20) and hyperosmotic response (GrpE), are important for stress tolerance [132, 133], was seen for MAG1 MAG4 and MAG9 (Fig. S24). Ammonia tolerance and resistance is a physiological phenomenon rather than a genetic property. Several complex mechanisms, i.e. osmo-tolerance, ionic membrane transport, molecular chaperones. *etc*. impart physiological resistance to evade metabolic deterioration under ammonia stress [132–135]. These stress genes have been found to be upregulated under acetate/acetic acid stress in *E. coli* [101]. Expression of stress-related proteins in MAG1, MAG4 and MAG9 could be due to the thermophilic temperature and high ammonia concentration applied in this study (Fig. S24). The transcriptomic response of stress-related genes of SPOB has not been characterised, so the exact role of these stress-regulated genes in MAG4 requires further investigation. Several genes in the candidate SPOB (MAG4), SAOB (MAG9) and the HM (MAG1), as also discussed above, were found to be present and expressed in an operon-like fashion (e.g. MMC, WLP, HM pathway genes, CoA-transferases, hydrogenases/dehydrogenases). The clustering of genes in SPOB (*Pelotomaculaceae* family, reviewed elsewhere [10]) has been proposed to enable energetically advantageous coordinated expression of series of genes, since it requires less transcriptional machinery [73, 136]. Similar to SPOB, the present results also indicate that the coordinated expression of series of genes energetically beneficial to ammonia-tolerant SAOB and methanogens.

## Conclusions

Use of a long-term enrichment approach to increase the abundance of an ammonia-tolerant syntrophic propionate-degrading community made it possible to identify key species and their metabolic activities, and to distinguish activities potentially related to the syntrophic lifestyle. Two novel ammonia-tolerant and thermophilic syntrophic species were identified, and we propose the name ‘*Candidatus* Thermosyntrophopropionicum ammoniitolerans’ for the SPOB. Batch cultivation, 16S rRNA gene analyses (sequencing and expression) and qPCR analysis indicated that *Methanothermobacter* could be crucial for syntrophic methanogenesis from propionate. Similar dependence for acetate degradation was not observed, indicating that the SAOB cooperated well with the *Methanoculleus* sp. also present in the syntrophic communities. Transcriptome data revealed activity related to sulphur metabolism, intercellular contact and molecular exchange by pili/flagellar appendages and nanotubes by the candidate SPOB, which can be crucial for efficient interdependent metabolism in a thermodynamically unfavourable environment. An additional bacterial species in the syntrophic community displayed activity for the reductive glycine pathway, but the wide substrate span of related bacterium and decreased abundance during cultivation without yeast extract suggest that this species is not directly involved in acid degradation. Thus, caution is needed when claiming that the reductive glycine pathway can be operated in the oxidative direction by SAOB to oxidise acetate. A deeper understanding of important syntrophic players and their mutualistic interactions under high-ammonia conditions is key for optimal design of anaerobic processes degrading protein-rich biomass. Future work should focus on identifying and characterising the functional interactions of ammonia-tolerant, thermophilic VFA-oxidising and methanogenic syntrophic communities as a model, which would be a milestone in metabolic modelling and systems biological approaches to anaerobic digester systems. With enhanced understanding of syntrophic synergy and coupled metabolic networks, industrial reactor operation can be steered to obtain higher efficiency and productivity of the methanogenic process.

### Supplementary information

Supplementary note

Supplementary figures

Supplementary tables

Supplementary data

## Data Availability

The data availability is described in Supplementary Note 7.
