# Supplementary material for: Syntrophic entanglements for propionate and acetate oxidation under thermophilic and high-ammonia conditions
Source: ISME J. 2023 Sep 7;17(11):1966–78. doi: 10.1038/s41396-023-01504-y (PMC10579422; doi:10.1038/s41396-023-01504-y)
Supplement: Supplementary file 1 — Supplementary note [file 41396_2023_1504_MOESM1_ESM.pdf]

Supplementary note

Supplementary note 1:

Nuclear Magnetic Resonance spectroscopy (NMR) was performed on a Bruker Avance III 600MHz spectrometer equipped with a QCI H-C/P/N-D cryoprobe and a SampleJet sample changer with a sample cooling system (Bruker Biospin AG, Fällanden, Switzerland). For acquisition and processing, TopSpin 3.5 supplied by Bruker was used, while Zgesgp was used for acquisition with a relaxation delay of 4 s and 128 scans for each experiment. All measurements were made at 25°C. After acquisition, each spectrum was Fourier-transformed after multiplication by a line broadening of 1.0 Hz, phase-corrected, baseline-corrected and referenced to total soluble protein (TSP) at 0.0 ppm. The spectra were then analysed using ChenomX NMR suite 7.5 (Chenomx Inc., Edmonton, Canada). The metabolites in each spectrum were identified using the Chenomx database and their concentrations were quantified to the internal standard TSP. Liquid samples (1 mL) were collected over the course of the incubation for DNA extraction and stored at -20°C until further analysis.

Supplementary note 2:

Temperature corrections for 52 °C versus 25 °C (the standard temperature) were made using the Gibbs-Helmholz equation, with H_2, CO_2, and CH_4 in the gas phase (standard conditions: partial pressure at 1 atm), and acetate in the aqueous phase (standard concentration at 1 M) [1]. The free energy (G_f) and enthalpy (H_f) values of the reactants and the products in their reference state were taken from Hanselmann (1991). Gibbs free energy (ΔG) values pertaining to actual concentrations and partial pressures were calculated using the Nernst equation, as described previously [1]. All calculation procedures are described in detail in protocols by Dolfing [3].

Supplementary note 3:

Total DNA was extracted from samples taken from reactors on 19 occasions (at 0, 15, 36, 57, 72, 86, 106, 120, 141, 159, 184, 198, 216, 248, 262, 286, 316, 433 and 450 days) and from each batch assay on 8-10 occasions (at 0, 4, 11, 18, 25, 32, 39, 46, 63 and 86 days). DNA extraction, construction of 16S rRNA gene amplicon libraries using primers 515F (GTGBCAGCMGCCGCGGTAA [4] and 805R (GACTACHVGGGTATCTAATCC [5], MiSeq Paired-end (2x300 bp) (Illumina) 16S rRNA gene amplicon sequencing was performed as described by Müller *et al*. [6] at Eurofins, Germany.

For whole metagenome sequencing only the samples from propionate enrichment reactors were used and samples from acetate enrichment reactors were excluded. Long read sequencing was done in house on a MinION device (Product code: MIN-101B, product description: MinION Mk1B, Oxford Nanopore Technologies) equipped with Spot-ON Flow Cell (R9.4.1) (Product code: FLO-MIN106D, Oxford Nanopore Technologies) using Ligation Sequencing Kit (SQK-LSK109, Oxford Nanopore Technologies) and Barcode Kit (EXP-NBD104, Oxford Nanopore Technologies). The long read sequencing software MinKNOW Core (v3.6.5) (Oxford Nanopore Technologies) running guppy base-caller (v4.0.15–1--bb42e40) (Oxford Nanopore Technologies) was used with base-calling on for 32 hours without barcode trimming which generated 1.89 M reads and 7.03 Giga bases. To replenish the flow cell in terms of sequencing speed and pore integrity re-fueling of flow cell with 70 µl of refuel mixture (35 µl nuclease free water and 35 µl sequencing buffer (SQB, LSK109 kit, Oxford Nanopore Technologies)) was done after 18 hr. Raw nanopore sequencing data were base-called and demultiplexed by guppy (v4.0.15–1--bb42e40) (Oxford Nanopore Technologies) on an inhouse GPU accelerated bioinformatics platform.

Supplementary note 4:

Shotgun metagenomic sequencing short read HiSeq data was quality controlled (Q-score > 20) and adapter trimming was performed using fastp [7]. Long read DNA genomic DNA extraction and library preparation was done using protocol described elsewhere [8]. For long read sequencing data quality control, sequence filtering was done using filtlong (v0.2.0) [9] with threshold of minimum read length 1,000 bp . Hybrid genome assembly was performed using

flye (v2.8) [10, 11], genome assembly reconstruction was conducted using racon (v1.4.13) [12] and resulting long read genome assembly was polished by medaka (v1.0.3) (Oxford Nanopore Technologies, 2018). Refinement of subsequent long read assembly was done with short reads using multiple polishing rounds with Pilon (v1.23) [13]. Metagenome assembled genomes (MAGs) were constructed with metaWRAP pipeline [14] which uses multiple binning algorithms, viz. metaBAT2 (v2.13) [15], CONCOCT (v1.1.0) [16], and MaxBin2 (v2.2.7) [17] for binning and bin refinements. The quality of bins in terms of completeness, contamination and strain heterogeneity was determined by CheckM (v1.0.18) [18]. In the present study, we used completeness >80% and contamination <15% as minimum criteria for a metagenomic bin to be described as MAG. A total of 58 metagenomic bins were generated which upon refinement and bin quality control resulted in 9 MAGs. These MAGs consisted of 8 bacterial and 1 archaeal MAGs. Functional annotations of bacterial MAGs was done with Bakta (v1.4.1) [19] using default parameters and while Prokka (v1.14.6) [20] was used for reference-based annotation of archaeal MAG (MAG1) with '*Candidatus* Methanoculleus thermohydrogenotrophicum' assembly (GCA_012521495.1) as reference. This reference was chosen for the functional annotation because the taxonomic annotation of archaeal MAG1 with GTDB-tk placed MAG1 in proximity to *Ca.* M. thermohydrogenotrophicum assembly (GCA_012521495.1) with 99% ANI. Furthermore, since *Ca.* M. thermohydrogenotrophicum assembly GCA_012521495.1 is not a representative assembly in GTDB, *Ca.* M. thermohydrogenotrophicum assembly GCA_001512375.1 was used for dDDH distance, ANI calculations and phylogenetic tree construction. Taxonomic profiling of methanogen from the metagenomics data was done using custom methanogen database formatted for kraken2 [21]. This database was created with all available representative genomics assemblies of genus in *Methanoculleus* and *Methanothermobacter* in GTDB.

Supplementary note 5:

The detailed information 16S rRNA gene amplicon sequencing generated data and its processing statistics for the enrichment reactors and batch assays is as follows:

1: Enrichment reactors

Out of 103 samples, on average 233,780 reads per sample were generated in 16S rRNA amplicon sequencing and 218,648 reads passed through the quality control/adapter trimming.

After reads processing with DADA2 algorithm 70,034 non-chimeric reads were finally used for the taxonomic annotations. Further, during the results visualization, the ASVs with unknown taxonomy at kingdom level were discarded finally resulting 11,056 ASVs (10230 bacterial and 826 archaeal ASVs).

2: Batch assays

There was a total of 100 samples from the batch assays with average 136,812 reads per sample. Quality filtering/adapter trimming yielded 124,175 reads per sample which were used for processing with DADA2 algorithm and 58,960 non-chimeric reads per sample were resulted and used for taxonomic annotations. A total of 5532 ASVs were annotated at kingdom level (5372 bacterial and 160 archaeal ASVs) and ASVs having unknown taxonomy at kingdom level were discarded.

Supplementary note 6:

MAG5 expressed genes encoding the reductive glycine pathway (rGlyP) and Wood-Ljungdahl pathway associated proteins. rGlyP has been described as a synthetic pathway for formate assimilation and shown to be used by the sulphate-reducing bacterium *Desulfovibrio desulphuricans* to generate pyruvate via CO_2 fixation or formate assimilation [22, 23]. Similarly to *D. desulphuricans*, MAG5 does not encode a complete WLP. A feature of interest for high-ammonia systems, is that the rate of rGlyP increases with increasing ammonia level [23]. The metabolic rGly route used by *D. desulphuricans* requires ATP, which is obtained via respiration with sulphate as electron acceptor [23]. However, sulphate was not present in our syntrophic cultures. Instead, as previously suggested, the bacteria could use similar energy-conserving mechanisms as in the WLP and operate rGlyP in combination with bifurcating enzymes to generate ATP [23]. It has also been proposed that rGlyP can be operated in the oxidative direction by SAOB to oxidise acetate (e.g. 89). Such a scenario would result in competition between MAG5 and MAG9 for acetate. However, the oxidative direction imposes thermodynamic constraints [25] and, given the lack of sound experimental evidence, this scenario should be interpreted as speculative. On the other hand, driving rGlyP in the reductive direction for consumption of formate (generated during fatty acid degradation by the SPOB and SAOB) would mean that the species produces acetate and potentially competes with the methanogen for formate [26]. Nonetheless, it is important to note that members of

Acetomicrobium can grow on a wide range of substrates, including organic acids, amino acids, dicarboxylic acids and other sugars, and that some species can reduce cysteine to sulphide [27–29]. Continuing cultivation of the syntrophic community in the present study demonstrated decreased abundance of *Acetomicrobium* on omitting yeast extract in the growth media (data not shown). Hence, MAG5 most likely fermented compounds included in the yeast extract in the culture investigated here, or possibly grew oxidatively using cysteine as electron acceptor.

Supplementary note 7:

The raw data for 16S rRNA gene amplicon sequencing from reactors and batch assays have been submitted to NCBI, under BioProject number PRJNA943381 and PRJNA943624, respectively. Raw sequence data from metagenomics sequencing (RP1, RP2) (Short read Illumina data and Long read Oxford Nanopore data) and MAGs are available in NCBI (BioProject PRJNA944114, MAG accession numbers: supplementary data). The MAG annotations generated and used in this study is available at the github repository https://github.com/abhijeetsingh1704/SPOB_SAOB_HM_MAGs. The raw metatranscriptomics data are available at NCBI under BioProject number PRJNA944124.

References

1. Dolfing J, Larter SR, Head IM. Thermodynamic constraints on methanogenic crude oil biodegradation. *ISME J* 2008; **2**: 442–452.

2. Hanselmann KW. Microbial energetics applied to waste repositories. *Experientia* 1991; **47**: 645–687.

3. Dolfing J. Protocols for Calculating Reaction Kinetics and Thermodynamics. In: McGenity TJ, Timmis KN, Nogales Fernández B (eds). *Hydrocarbon and Lipid Microbiology Protocols: Statistics, Data Analysis, Bioinformatics and Modelling*. 2016. Springer Berlin Heidelberg, Berlin, Heidelberg, pp 155–163.

4. Hugerth LW, Wefer HA, Lundin S, Jakobsson HE, Lindberg M, Rodin S, et al. DegePrime, a program for degenerate primer design for broad-taxonomic-range PCR in microbial ecology studies. *Appl Environ Microbiol* 2014; **80**: 5116–5123.

5. Herlemann DPR, Labrenz M, Jürgens K, Bertilsson S, Waniek JJ, Andersson AF. Transitions in bacterial communities along the 2000 km salinity gradient of the Baltic Sea. *ISME J* 2011; **5**: 1571–1579.

6. Müller B, Sun L, Westerholm M, Schnürer A. Bacterial community composition and *fhs* profiles of low- and high-ammonia biogas digesters reveal novel syntrophic acetate-oxidising bacteria. *Biotechnol Biofuels* 2016; **9**: 48.

7. Chen S, Zhou Y, Chen Y, Gu J. fastp: an ultra-fast all-in-one FASTQ preprocessor. *Bioinformatics* 2018; **34**: i884–i890.

8. Schnürer A, Singh A, Bi S, Qiao W, Westerholm M. *Miniphocaeibacter halophilus* sp. nov., an ammonium-tolerant acetate-producing bacterium isolated from a biogas system. *Int J Syst Evol Microbiol* 2022; **72**: 1466–5034.

9. Wick R. Filtlong: tool for filtering long reads by quality. 2017. Available at: https://github.com/rrwick/Filtlong.

10. Kolmogorov M, Yuan J, Lin Y, Pevzner PA. Assembly of long, error-prone reads using repeat graphs. *Nat Biotechnol* 2019; **37**: 540–546.

11. Lin Y, Yuan J, Kolmogorov M, Shen MW, Chaisson M, Pevzner PA. Assembly of long error-prone reads using de Bruijn graphs. *Proc Natl Acad Sci* 2016; **113**.

12. Vaser R, Sović I, Nagarajan N, Šikić M. Fast and accurate de novo genome assembly from long uncorrected reads. *Genome Res* 2017; **27**: 737–746.

13. Walker BJ, Abeel T, Shea T, Priest M, Abouelliel A, Sakthikumar S, et al. Pilon: An Integrated Tool for Comprehensive Microbial Variant Detection and Genome Assembly Improvement. *PLoS One* 2014; **9**: e112963.

14. Uritskiy G V., DiRuggiero J, Taylor J. MetaWRAP—a flexible pipeline for genome-resolved metagenomic data analysis. *Microbiome* 2018; **6**: 158.

15. Kang DD, Froula J, Egan R, Wang Z. MetaBAT, an efficient tool for accurately reconstructing single genomes from complex microbial communities. *PeerJ* 2015; **3**: e1165.

16. Alneberg J, Bjarnason BS, de Bruijn I, Schirmer M, Quick J, Ijaz UZ, et al. Binning metagenomic contigs by coverage and composition. *Nat Methods* 2014; **11**: 1144–

1146.

17. Wu Y-W, Tang Y-H, Tringe SG, Simmons BA, Singer SW. MaxBin: an automated binning method to recover individual genomes from metagenomes using an expectation-maximization algorithm. *Microbiome* 2014; **2**: 26.

18. Parks DH, Imelfort M, Skennerton CT, Hugenholtz P, Tyson GW. CheckM: assessing the quality of microbial genomes recovered from isolates, single cells, and metagenomes. *Genome Res* 2015; **25**: 1043–1055.

19. Schwengers O, Jelonek L, Dieckmann MA, Beyvers S, Blom J, Goesmann A. Bakta: rapid and standardized annotation of bacterial genomes via alignment-free sequence identification. *Microb Genomics* 2021; **7**: 000685.

20. Seemann T. Prokka: rapid prokaryotic genome annotation. *Bioinformatics* 2014; **30**: 2068–2069.

21. Wood DE, Lu J, Langmead B. Improved metagenomic analysis with Kraken 2. *Genome Biol* 2019; **20**: 257.

22. Bar-Even A, Noor E, Flamholz A, Milo R. Design and analysis of metabolic pathways supporting formatotrophic growth for electricity-dependent cultivation of microbes. *Biochim Biophys Acta - Bioenerg* 2013; **1827**: 1039–1047.

23. Sánchez-Andrea I, Guedes IA, Hornung B, Boeren S, Lawson CE, Sousa DZ, et al. The reductive glycine pathway allows autotrophic growth of *Desulfovibrio desulfuricans*. *Nat Commun* 2020; **11**: 5090.

24. Zhu X, Campanaro S, Treu L, Seshadri R, Ivanova N, Kougias PG, et al. Metabolic dependencies govern microbial syntrophies during methanogenesis in an anaerobic digestion ecosystem. *Microbiome* 2020; **8**: 22.

25. Bar-Even A, Noor E, Milo R. A survey of carbon fixation pathways through a quantitative lens. *J Exp Bot* 2012; **63**: 2325–2342.

26. Thiele JH, Zeikus JG. Control of Interspecies Electron Flow during Anaerobic Digestion: Significance of Formate Transfer versus Hydrogen Transfer during Syntrophic Methanogenesis in Flocs. *Appl Environ Microbiol* 1988; **54**: 20–29.

27. Rees GN, Patel BKC, Grassia GS, Sheehy AJ. *Anaerobaculum thermoterrenum* gen.

nov., sp. nov., a Novel, Thermophilic Bacterium Which Ferments Citrate. *Int J Syst Bacteriol* 1997; **47**: 150–154.

28. Hania W Ben, Bouanane-Darenfed A, Cayol J-L, Ollivier B, Fardeau M-L. Reclassification of *Anaerobaculum mobile*, *Anaerobaculum thermoterrenum*, *Anaerobaculum hydrogeniformans* as *Acetomicrobium mobile* comb. nov., *Acetomicrobium thermoterrenum* comb. nov. and *Acetomicrobium hydrogeniformans*. *Int J Syst Evol Microbiol* 2016; **66**: 1506–1509.

29. Menes RJ, Muxí L. *Anaerobaculum mobile* sp. nov., a novel anaerobic, moderately thermophilic, peptide-fermenting bacterium that uses crotonate as an electron acceptor, and emended description of the genus *Anaerobaculum*. *Int J Syst Evol Microbiol* 2002; **52**: 157–164.
