# Supplementary material for: Syntrophic entanglements for propionate and acetate oxidation under thermophilic and high-ammonia conditions
Source: ISME J. 2023 Sep 7;17(11):1966–78. doi: 10.1038/s41396-023-01504-y (PMC10579422; doi:10.1038/s41396-023-01504-y)
Supplement: Supplementary file 2 — Supplementary figures [file 41396_2023_1504_MOESM2_ESM.pdf]

Supplementary Figures

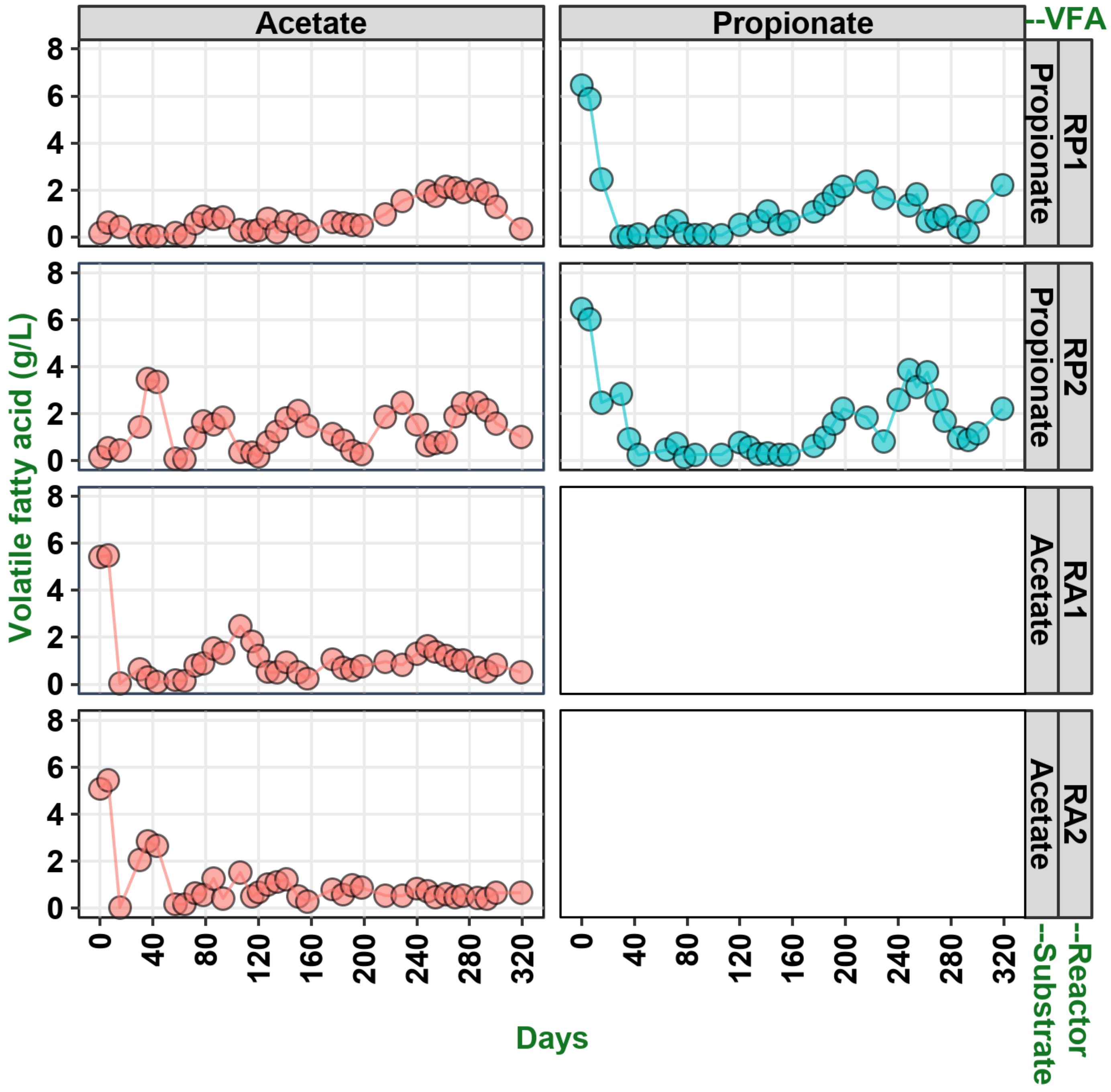

Figure S1. Volatile fatty acid (acetate, propionate) levels in acetate-fed (RA1, RA2) and propionate-fed (RP1, RP2) reactors over the course of the operating period.

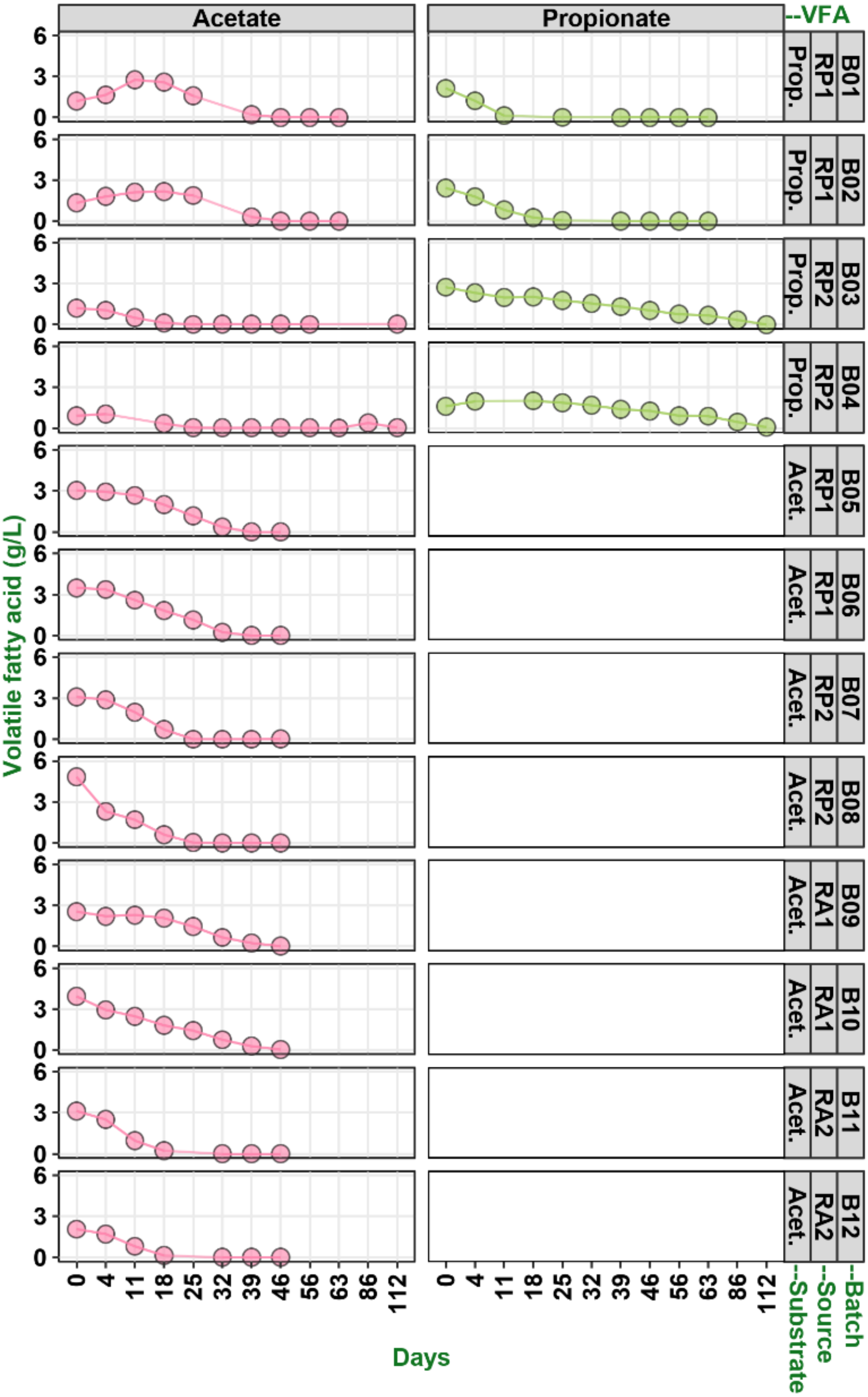

Figure S2. Concentration (g L^{-1}) of the volatile fatty acids (VFA) acetate and propionate in batch assays with propionate (B01-B04) and acetate (B05-B12) as the primary growth substrate.

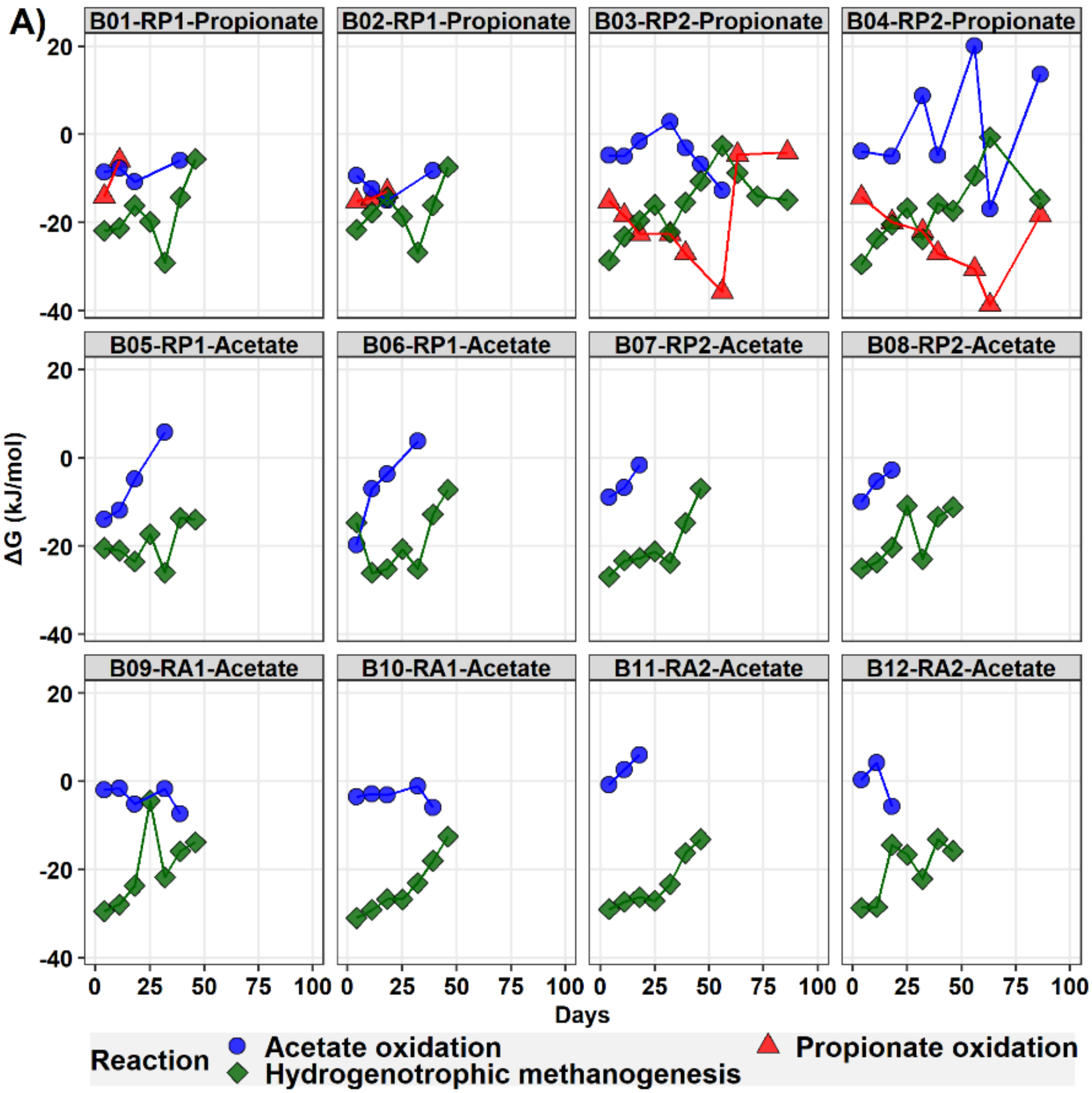

A)
B01-RP1-Propionate
B02-RP1-Propionate
B03-RP2-Propionate
B04-RP2-Propionate
B05-RP1-Acetate
B06-RP1-Acetate
B07-RP2-Acetate
B08-RP2-Acetate
B09-RA1-Acetate
B10-RA1-Acetate
B11-RA2-Acetate
B12-RA2-Acetate
ΔG (kJ/mol)
20
0
-20
-40
0 25 50 75 100
Days
Reaction
Acetate oxidation
Hydrogenotrophic methanogenesis
Propionate oxidation

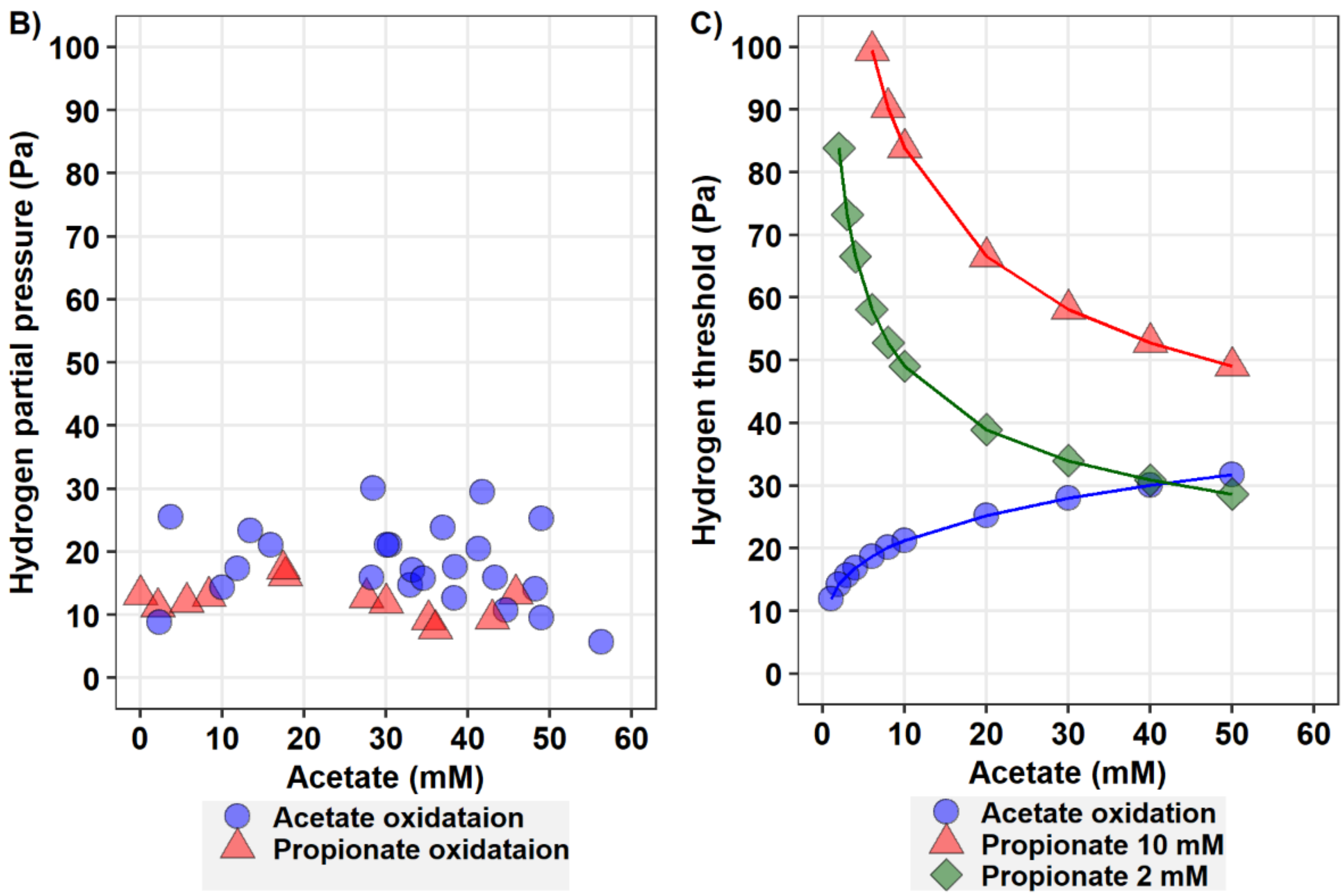

Figure S3. A) Change in Gibbs free energy (ΔG, kJ mol^{-1}) over the course of batch assays for acetoclastic methanogenesis, hydrogenotrophic methanogenesis, syntrophic acetate oxidation and syntrophic propionate oxidation-mediated methanogenesis. **B)** The hydrogen threshold for the SPOB and SAOB in relation to different acetate concentration. **C)** The combination of acetate and hydrogen levels observed in the batch experiments during degradation of acetate and propionate.

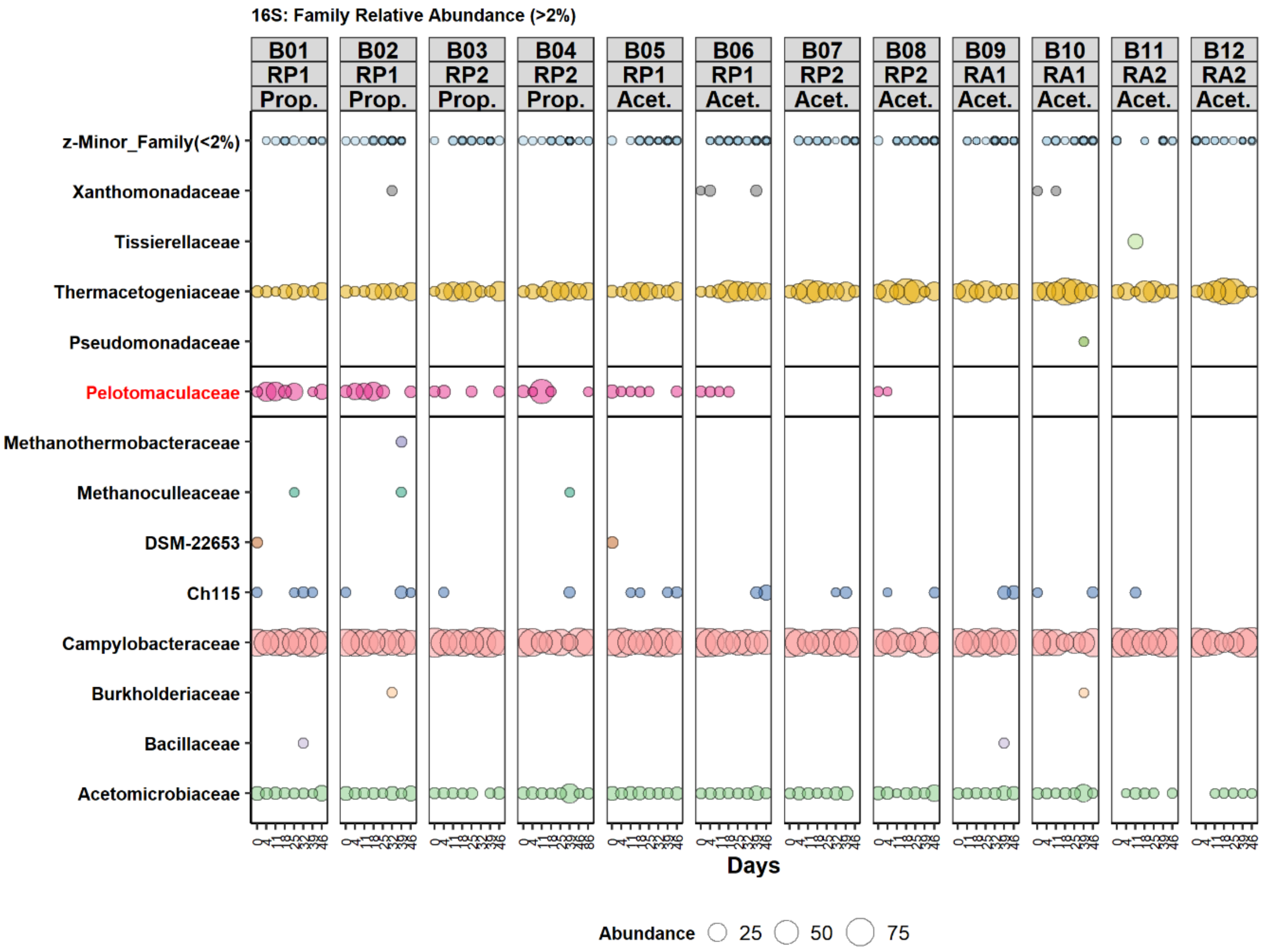

Figure S4. Bubble plot showing percentage relative abundance (>2%) of microbial communities at family level in the batch assays with propionate (B01-B04) and acetate (B05-B12) as the primary growth substrate.

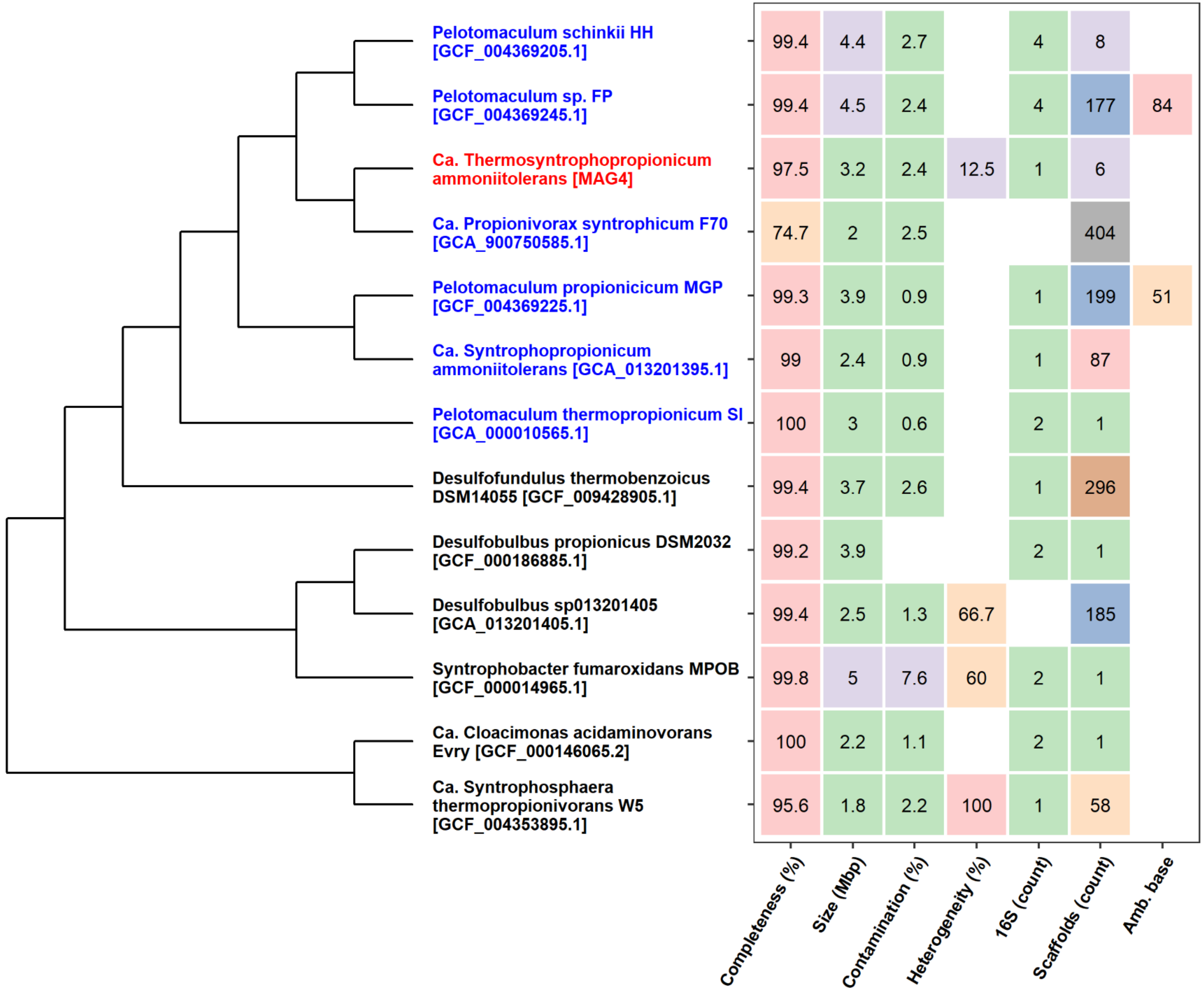

Figure S5. Species tree based on the genome orthologous sequences of **MAG4** and other related SPOBs. Species and candidates in blue belong to the family *Pelotomaculaceae*.

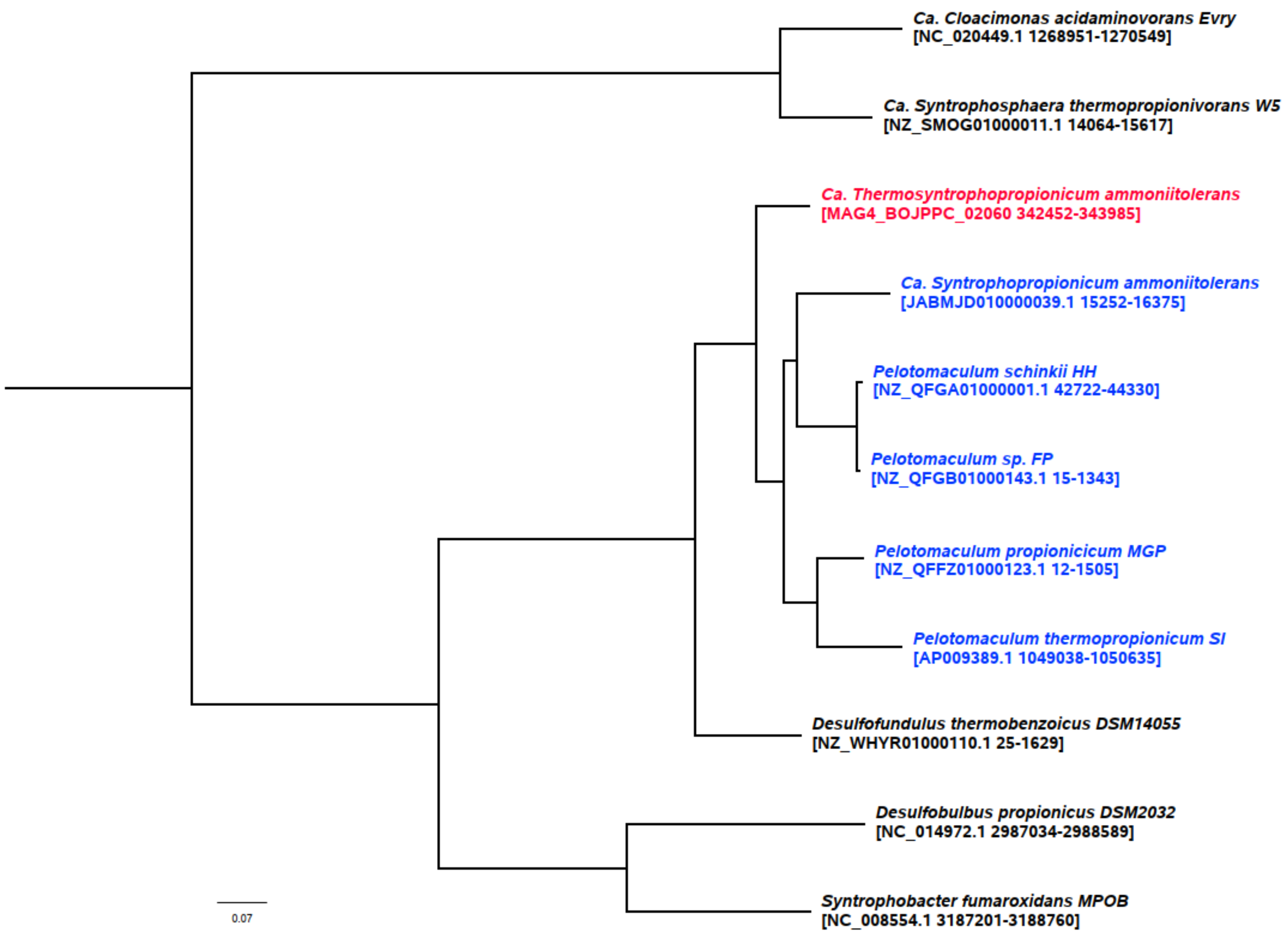

Figure S6. Gene tree based on the 16S rRNA gene sequences of **MAG4** and other related SPOBs. Species and candidates in blue belong to the family *Pelotomaculaceae*.

a)

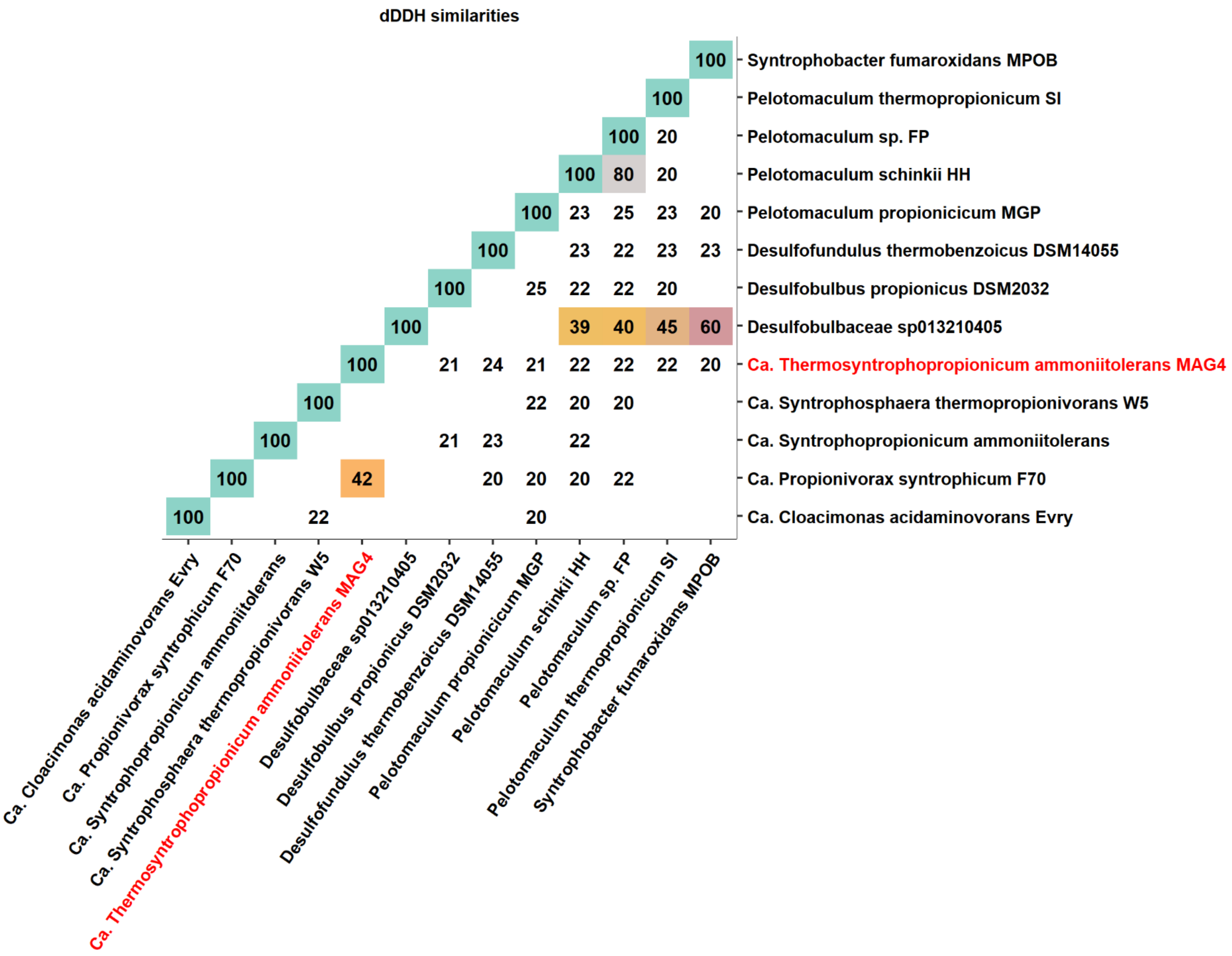
dDDH similarities
Syntrophobacter fumaroxidans MPOB
Pelotomaculum thermopropionicum SI
Pelotomaculum sp. FP
Pelotomaculum schinkii HH
Pelotomaculum propionicicum MGP
Desulfofundulus thermobenzoicus DSM14055
Desulfobulbus propionicus DSM2032
Desulfobulbaceae sp013210405
Ca. Thermosyntrophopropionicum ammoniitolerans MAG4
Ca. Syntrophosphaera thermopropionivorans W5
Ca. Syntrophopropionicum ammoniitolerans
Ca. Propionivorax syntrophicum F70
Ca. Cloacimonas acidaminovorans Evry
100
100
100 20
100 80 20
100 23 25 23 20
100 23 22 23 23
100 25 22 22 20
100 39 40 45 60
100 21 24 21 22 22 22 20
100 22 20 20
100 21 23 22
100 42 20 20 20 22
100 22 20

b)

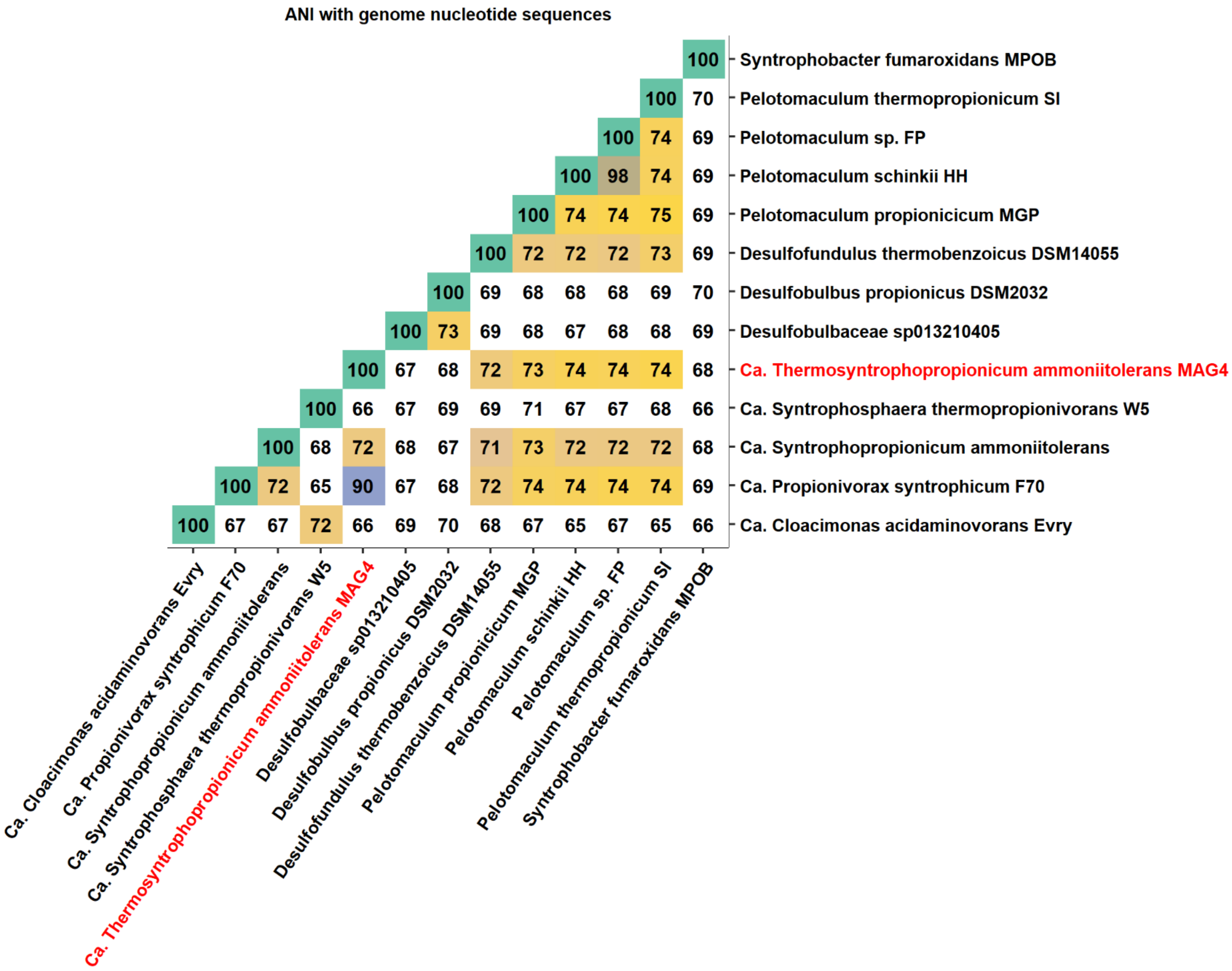
ANI with genome nucleotide sequences
100 Syntrophobacter fumaroxidans MPOB
100 70 Pelotomaculum thermopropionicum SI
100 74 69 Pelotomaculum sp. FP
100 98 74 69 Pelotomaculum schinkii HH
100 74 74 75 69 Pelotomaculum propionicicum MGP
100 72 72 72 73 69 Desulfofundulus thermobenzoicus DSM14055
100 69 68 68 68 69 70 Desulfobulbus propionicus DSM2032
100 73 69 68 67 68 68 69 Desulfobulbaceae sp013210405
100 67 68 72 73 74 74 74 68 Ca. Thermosyntrophopropionicum ammoniitolerans MAG4
100 66 67 69 69 71 67 67 68 66 Ca. Syntrophosphaera thermopropionivorans W5
100 68 72 68 67 71 73 72 72 72 68 Ca. Syntrophopropionicum ammoniitolerans
100 72 65 90 67 68 72 74 74 74 74 69 Ca. Propionivorax syntrophicum F70
100 67 67 72 66 69 70 68 67 65 67 65 66 Ca. Cloacimonas acidaminovorans Evry
Ca. Cloacimonas acidaminovorans Evry
Ca. Propionivorax syntrophicum F70
Ca. Syntrophopropionicum ammoniitolerans
Ca. Syntrophosphaera thermopropionivorans W5
Ca. Thermosyntrophopropionicum ammoniitolerans MAG4
Desulfobulbaceae sp013210405
Desulfobulbus propionicus DSM2032
Desulfofundulus thermobenzoicus DSM14055
Pelotomaculum propionicicum MGP
Pelotomaculum schinkii HH
Pelotomaculum sp. FP
Pelotomaculum thermopropionicum SI
Syntrophobacter fumaroxidans MPOB

c)

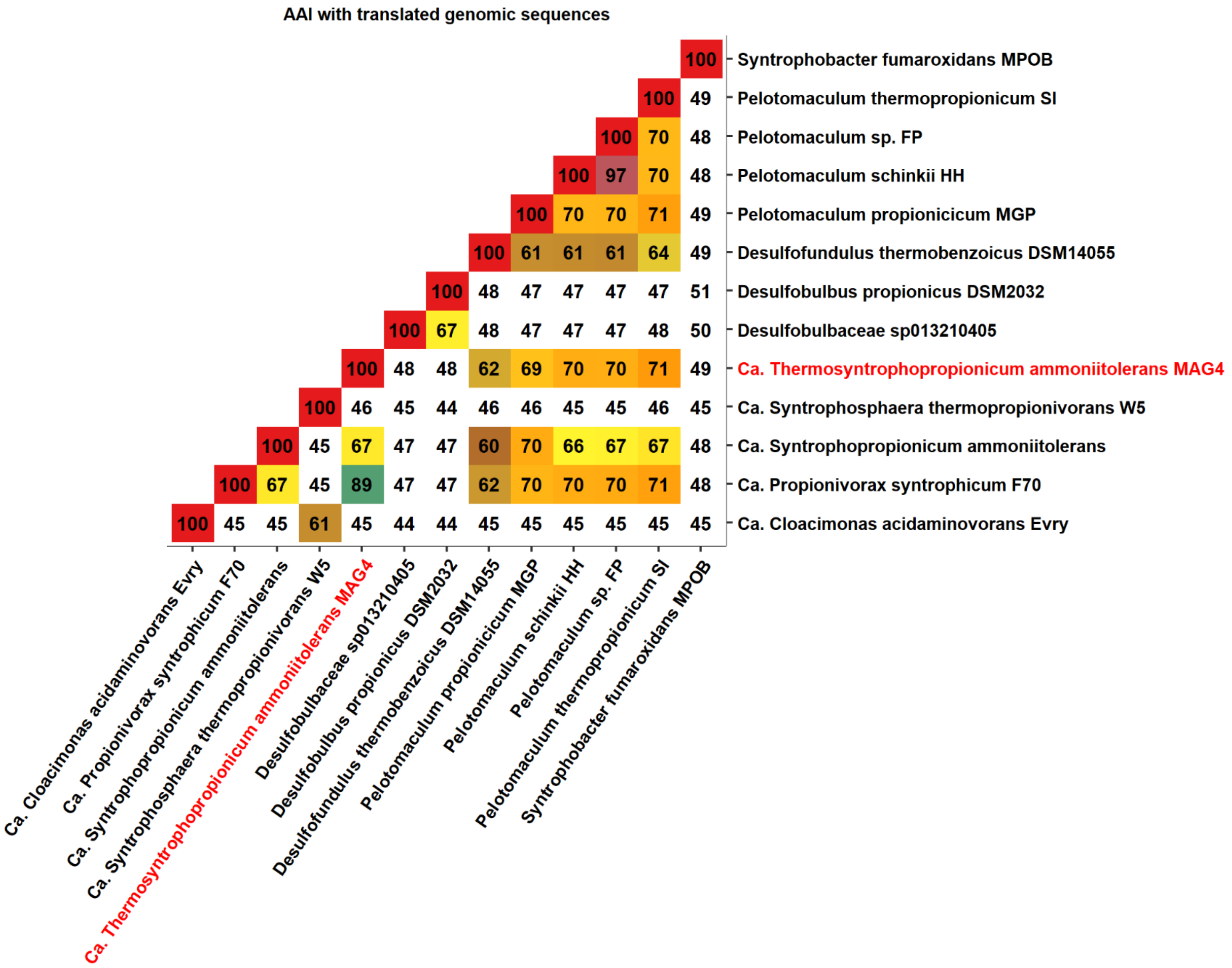

Figure S7. Comparison of genome similarities of **MAG4** with other known or proposed SPOB based on **a)** digital DNA-DNA hybridisation (dDDH), **b)** average nucleotide identity (ANI) and **c)** average amino acid identity (AAI).

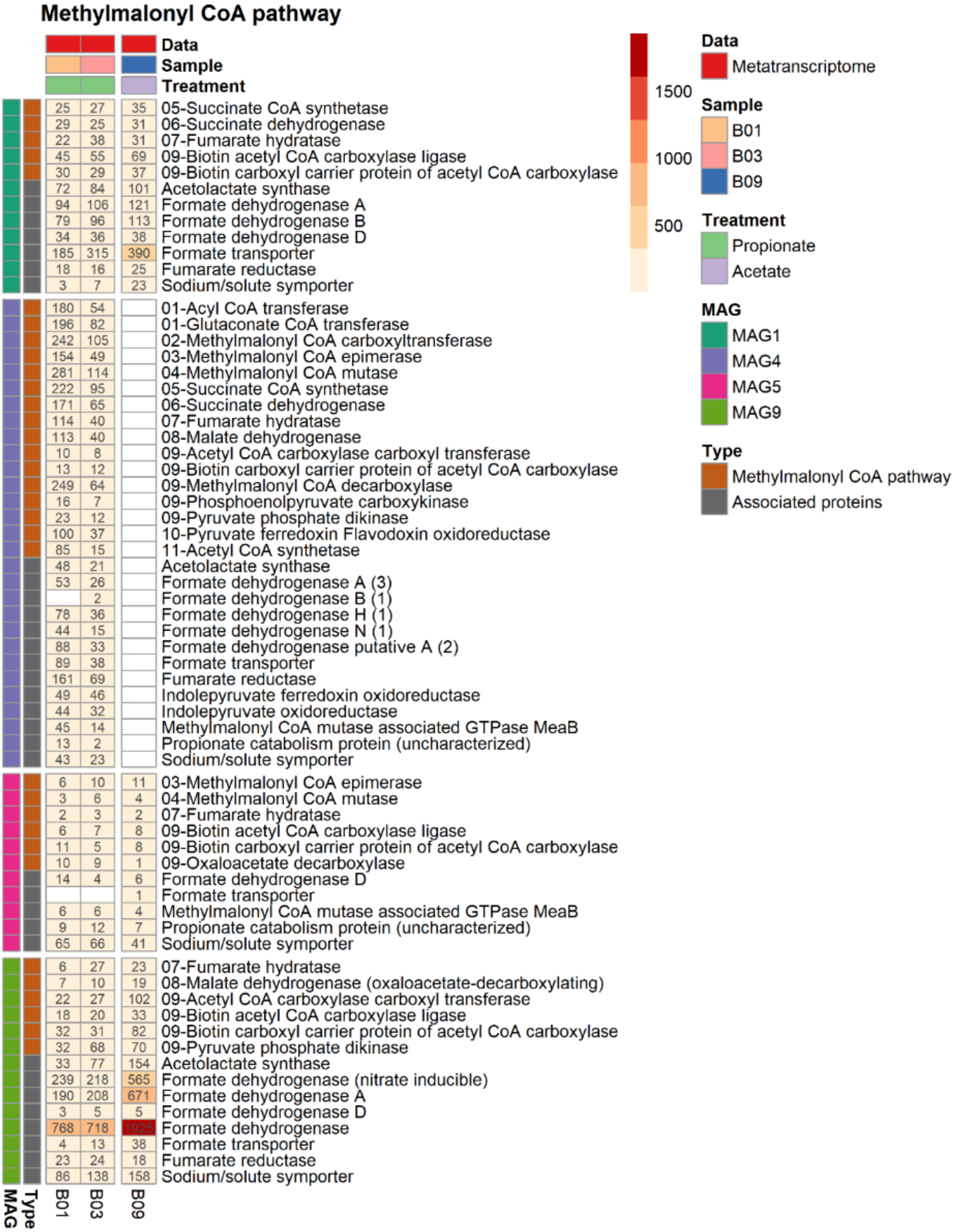

Figure S8. Metatranscriptomics expression profile of the methylmalonyl CoA (MMC) pathway of propionate metabolism (based on transcripts per million (TPM) counts) in propionate (B01 - faster propionate degradation, B03 - slower propionate degradation) versus acetate batch assay (B09) for the metagenomic assembled genomes (MAGs) other than the candidate syntrophic propionate-oxidising bacterium MAG4 identified by metagenomics analysis in the present study. The numerical values with the enzyme name denote the steps in MMC pathway.

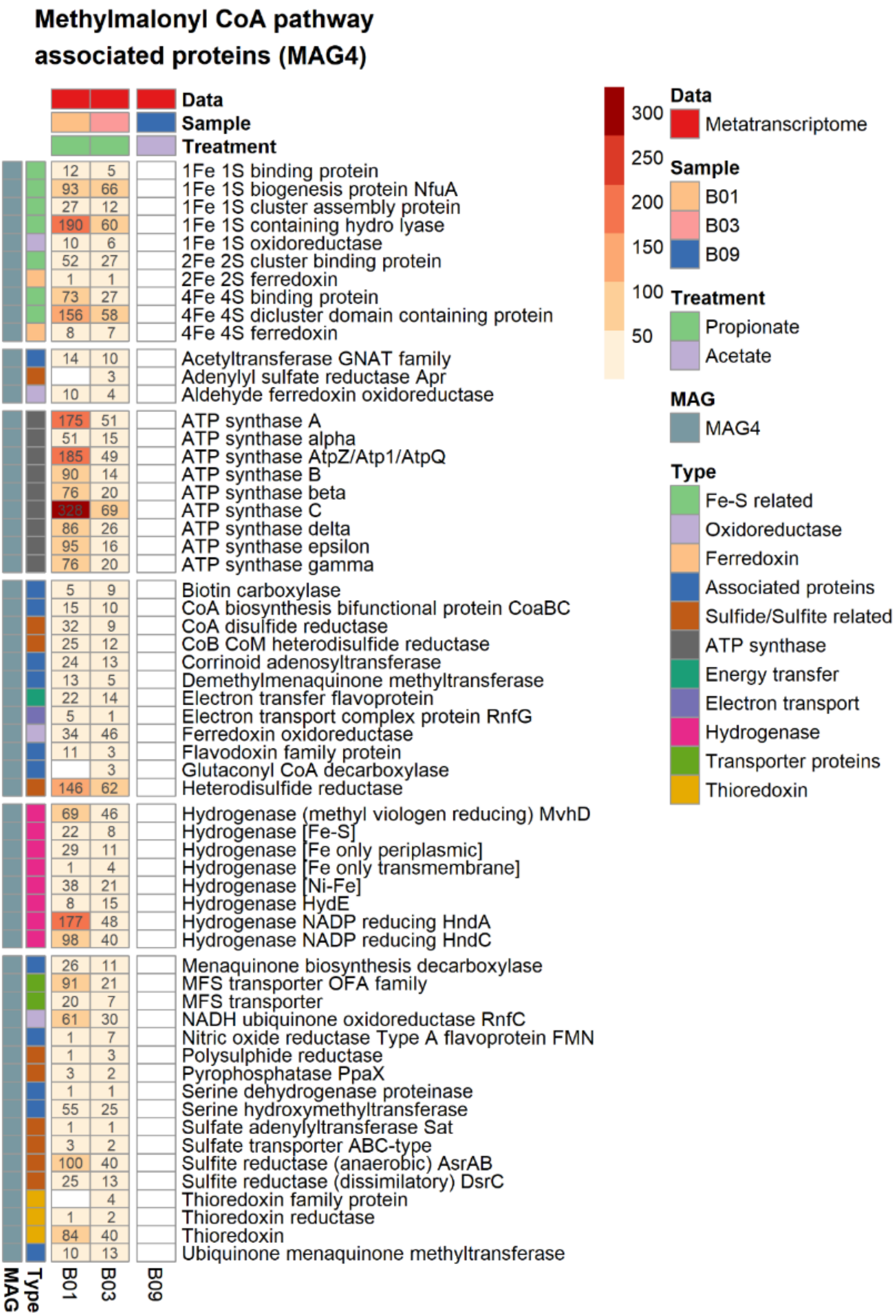

Figure S9. Metatranscriptomics expression profile of methylmalonyl CoA (MMC) pathway-associated proteins (based on transcripts per million (TPM) counts) for the metagenomic assembled genome (MAG) of the candidate syntrophic propionate-oxidising bacterium **MAG4**. The profiles show expression of genes in addition to genes presented in Fig 2 in propionate (B01 - faster propionate degradation, B03 - slower propionate degradation) versus acetate batch assay (B09) identified by metagenomics analysis in the present study.

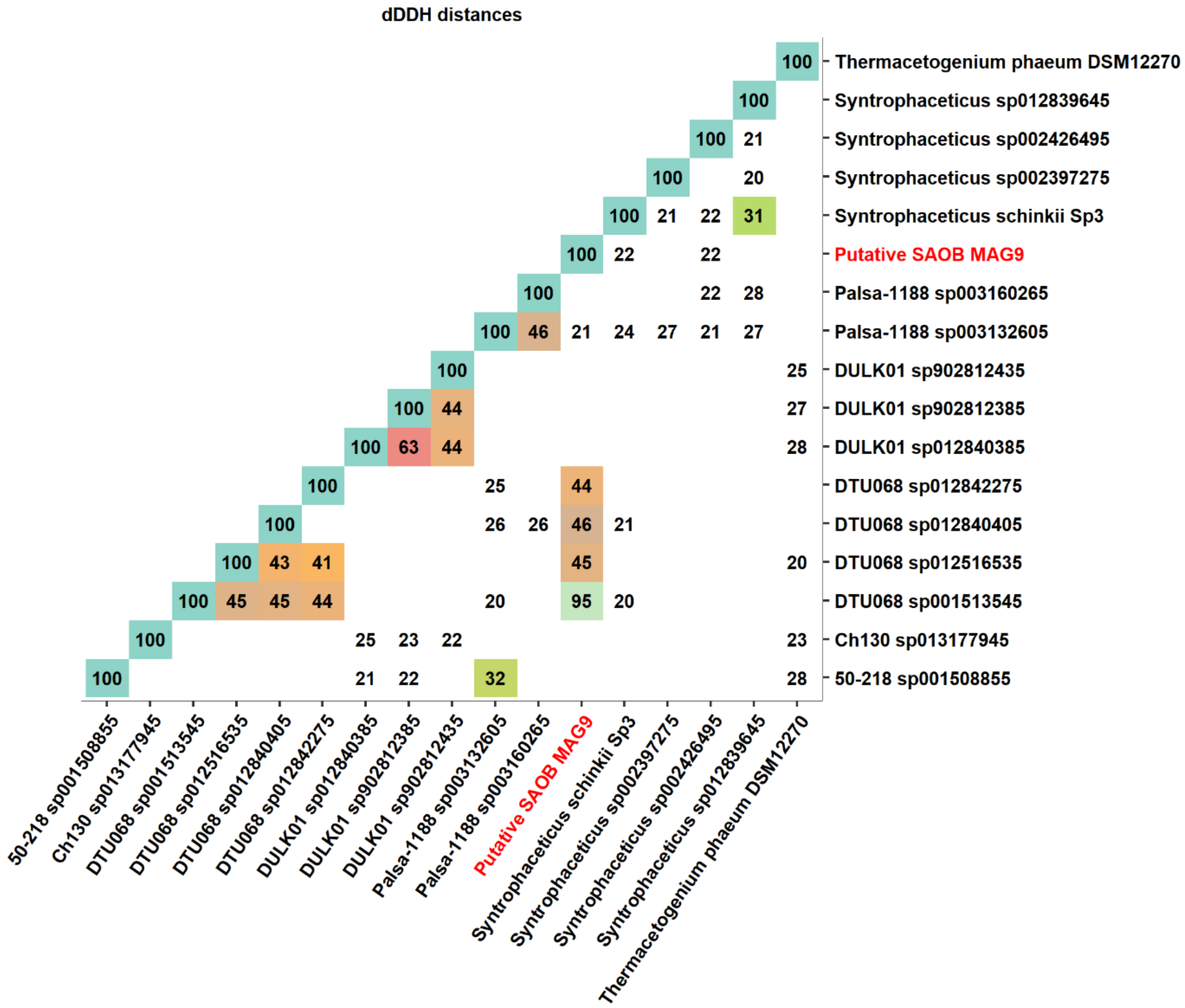

Figure S10. Comparison of genome similarities of **MAG9** with syntrophic acetate-oxidising bacteria (SAOB) and other species and candidates belonging to the family *Thermacetogeniaceae* based on digital DNA-DNA hybridisation (dDDH).

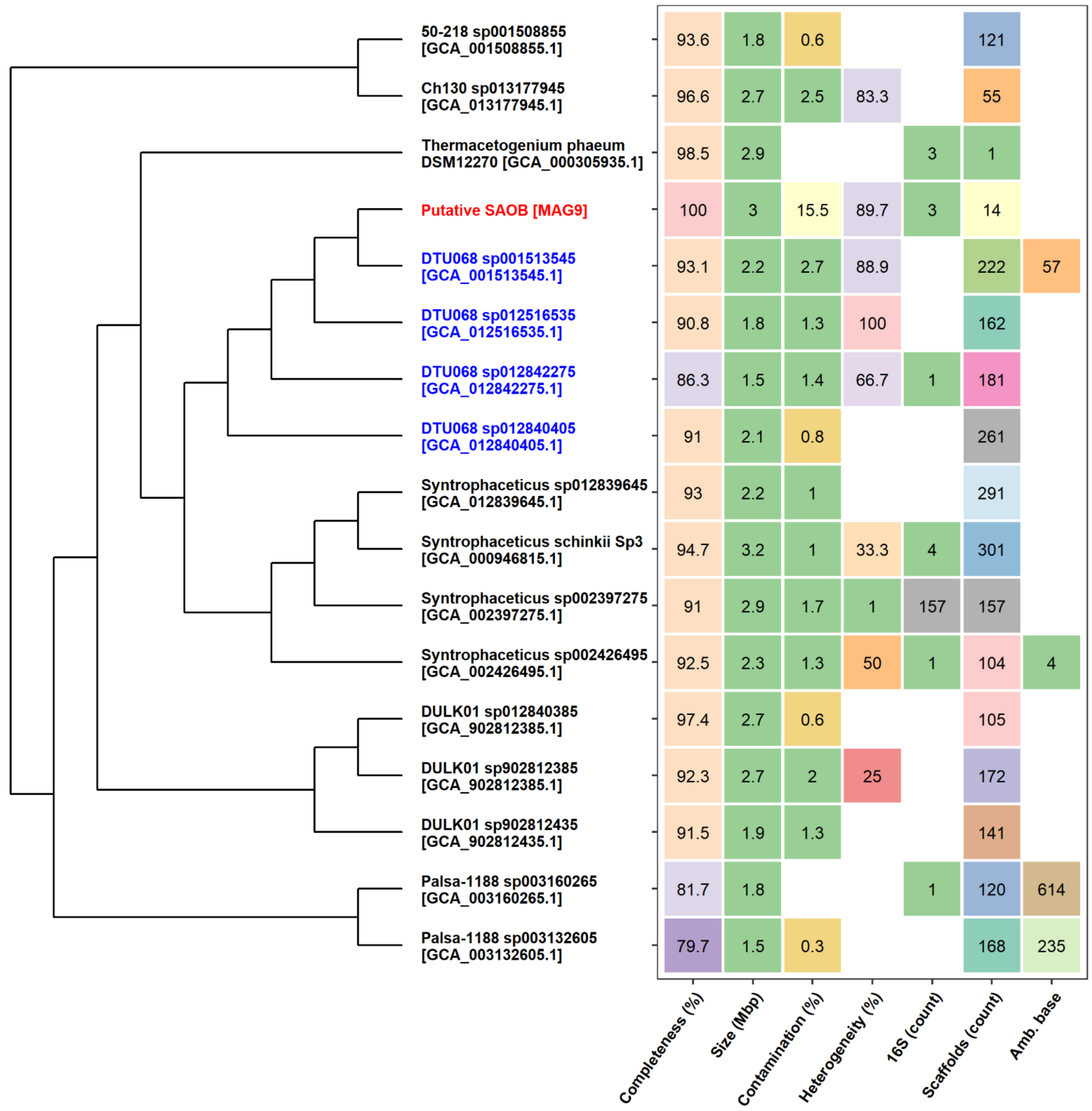

Species	Completeness (%)	Size (Mbp)	Contamination (%)	Heterogeneity (%)	16S (count)	Scaffolds (count)	Amb. base
50-218 sp001508855 [GCA_001508855.1]	93.6	1.8	0.6			121	
Ch130 sp013177945 [GCA_013177945.1]	96.6	2.7	2.5	83.3		55	
Thermacetogenium phaeum DSM12270 [GCA_000305935.1]	98.5	2.9			3	1	
Putative SAOB [MAG9]	100	3	15.5	89.7	3	14	
DTU068 sp001513545 [GCA_001513545.1]	93.1	2.2	2.7	88.9		222	57
DTU068 sp012516535 [GCA_012516535.1]	90.8	1.8	1.3	100		162	
DTU068 sp012842275 [GCA_012842275.1]	86.3	1.5	1.4	66.7	1	181	
DTU068 sp012840405 [GCA_012840405.1]	91	2.1	0.8			261	
Syntrophaceticus sp012839645 [GCA_012839645.1]	93	2.2	1			291	
Syntrophaceticus schinkii Sp3 [GCA_000946815.1]	94.7	3.2	1	33.3	4	301	
Syntrophaceticus sp002397275 [GCA_002397275.1]	91	2.9	1.7	1	157	157	
Syntrophaceticus sp002426495 [GCA_002426495.1]	92.5	2.3	1.3	50	1	104	4
DULK01 sp012840385 [GCA_902812385.1]	97.4	2.7	0.6			105	
DULK01 sp902812385 [GCA_902812385.1]	92.3	2.7	2	25		172	
DULK01 sp902812435 [GCA_902812435.1]	91.5	1.9	1.3			141	
Palsa-1188 sp003160265 [GCA_003160265.1]	81.7	1.8			1	120	614
Palsa-1188 sp003132605 [GCA_003132605.1]	79.7	1.5	0.3			168	235

Figure S11. Species tree based on the orthologous sequences of **MAG9**, syntrophic acetate-oxidising bacteria (SAOB) and other species and candidates belonging to the family *Thermacetogeniaceae*.

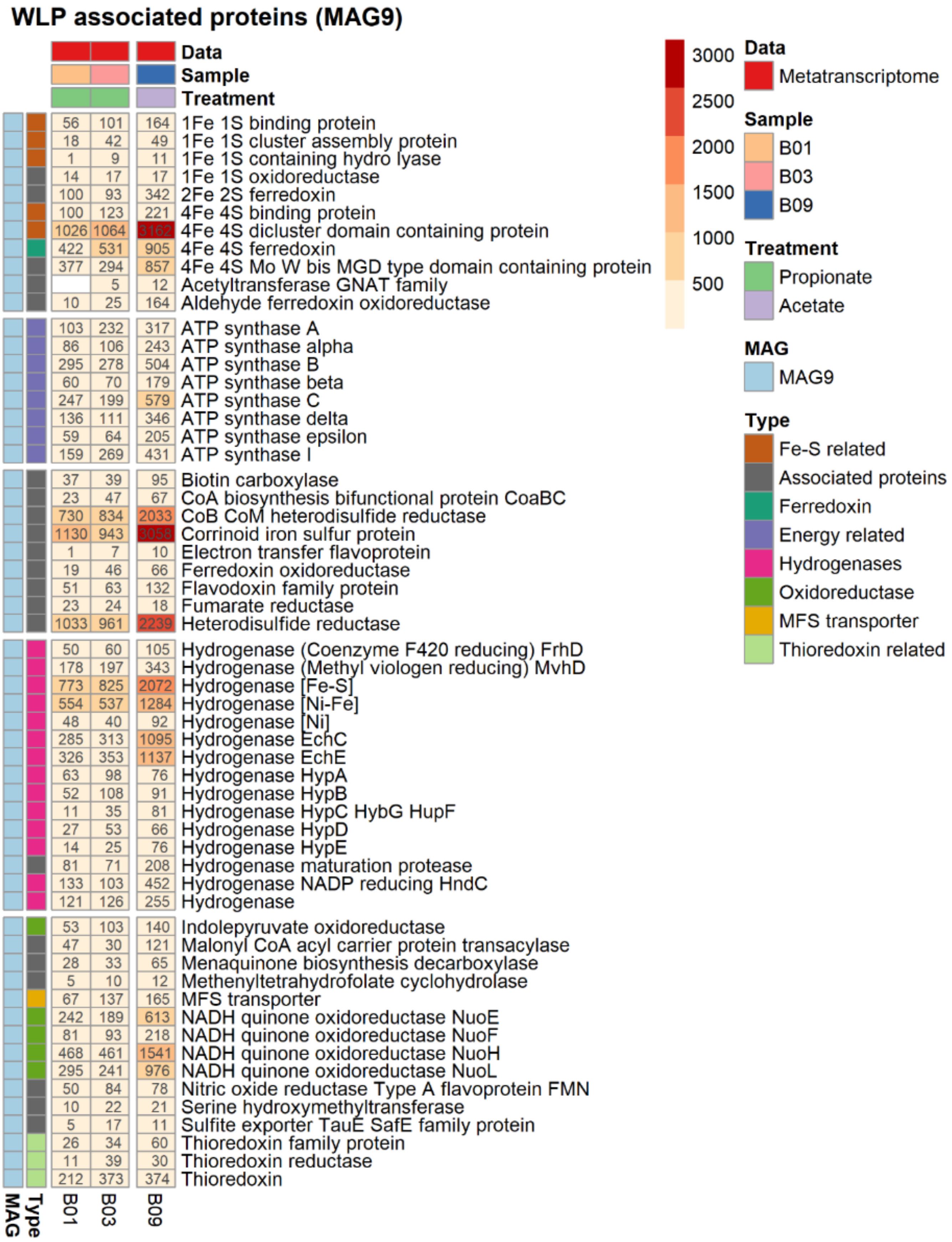

Figure S12. Metatranscriptomics expression profile of Wood-Ljungdahl pathway-associated proteins (based on transcripts per million (TPM) counts) for the metagenomic assembled genome (MAG) of the candidate syntrophic acetate-oxidising bacterium **MAG9**. The profiles show expression of genes identified by metagenomics analysis in addition to genes presented in Fig. 3 in propionate (B01 - faster propionate degradation, B03 - slower propionate degradation) versus acetate batch assay (B09).

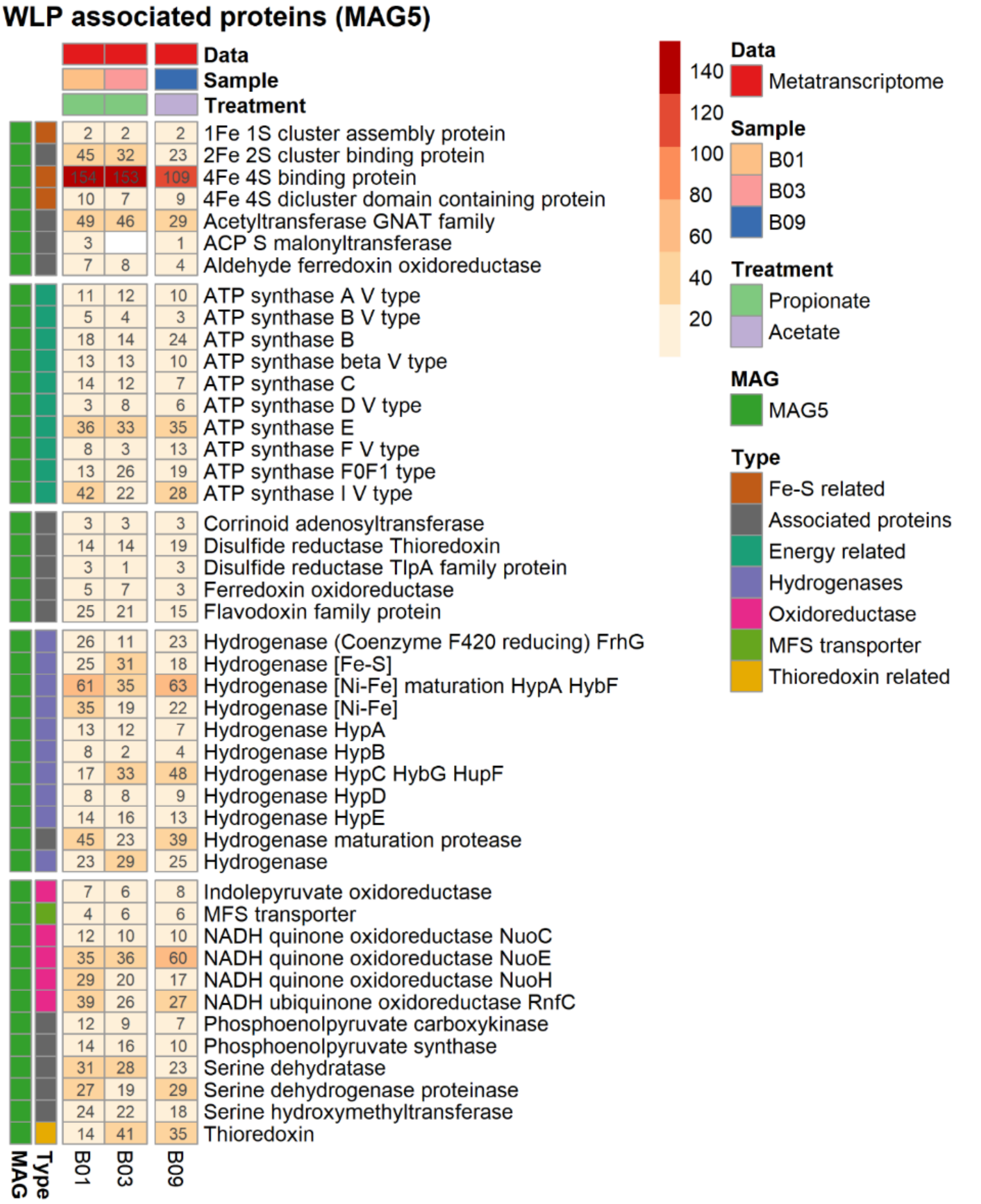

Figure S13. Metatranscriptomics expression profile of Wood-Ljungdahl pathway-associated proteins (based on transcripts per million (TPM) counts) for the metagenomic assembled genome (MAG) of the candidate syntrophic acetate-oxidising bacterium **MAG5**. The profiles show expression of genes identified by metagenomics analysis in addition to genes presented in Fig. 3 in propionate (B01 - faster propionate degradation, B03 - slower propionate degradation) versus acetate batch assay (B09).

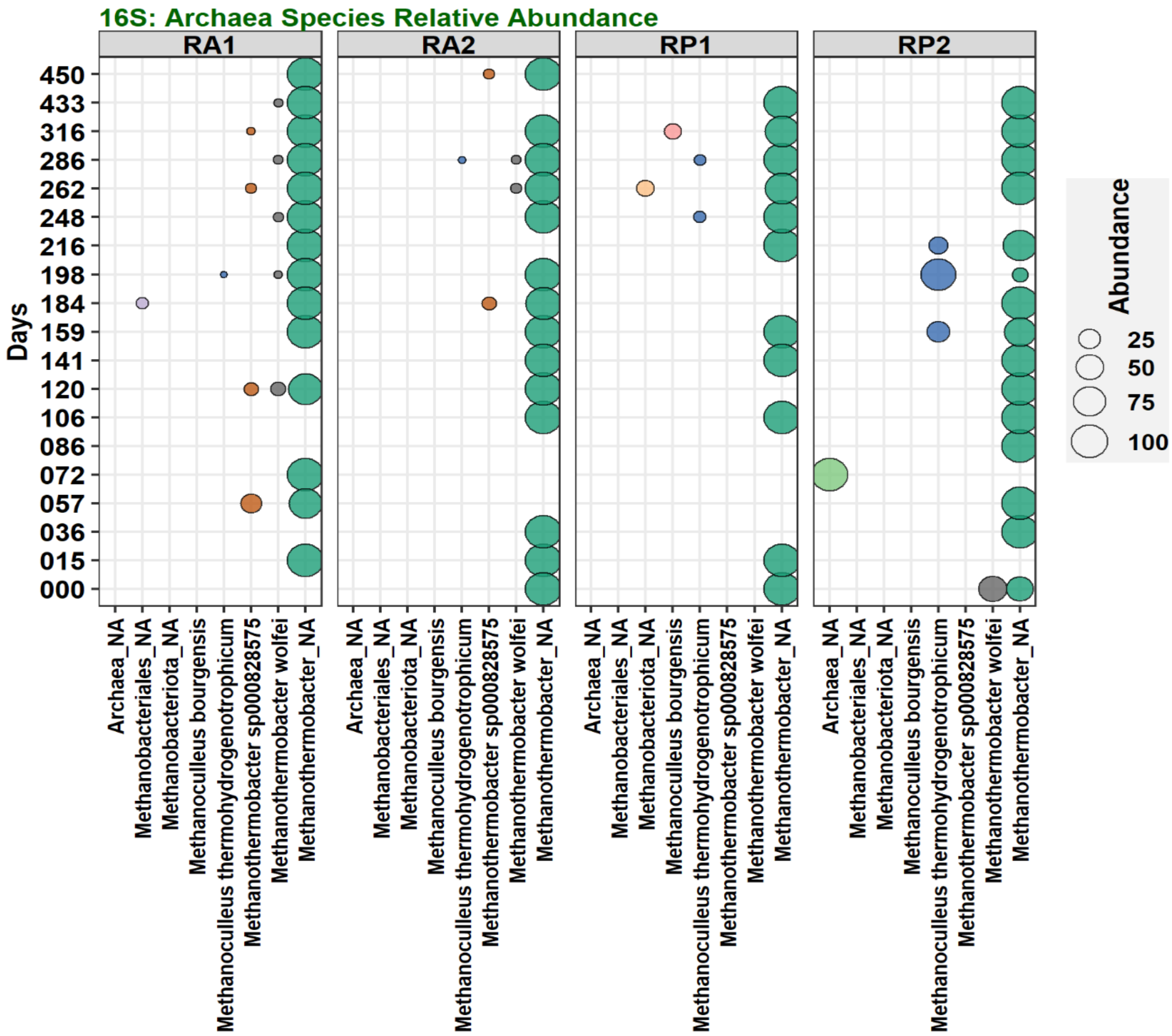

Figure S14. Relative abundance of methanogens in 16S rRNA gene amplicon sequencing based on total archaeal sequences in reactors fed acetate (RA1, RA2) and propionate (RP1, RP2).

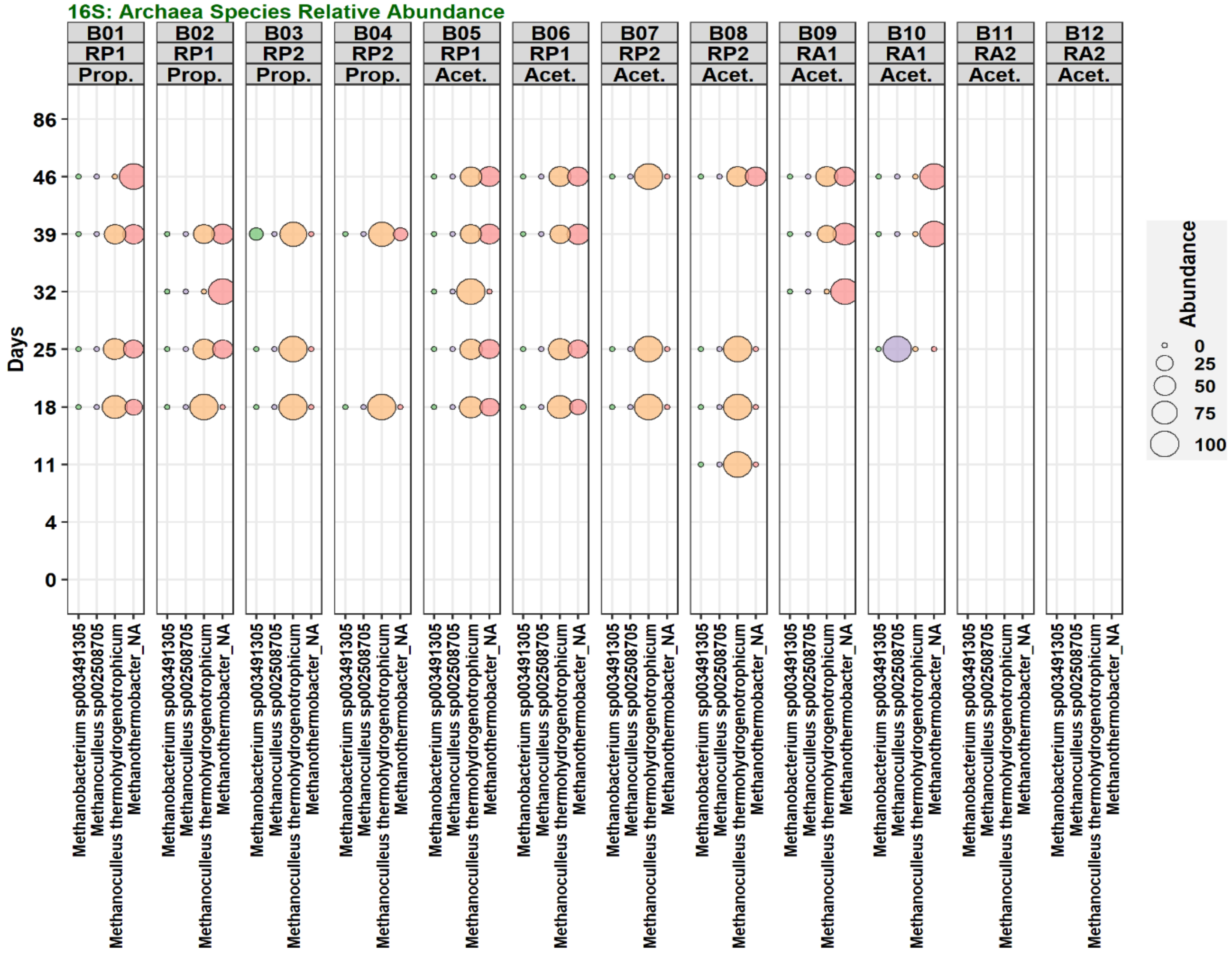

Figure S15. Relative abundance of methanogens of total archaeal sequences in 16S rRNA gene amplicon sequencing for samples from batch assays.

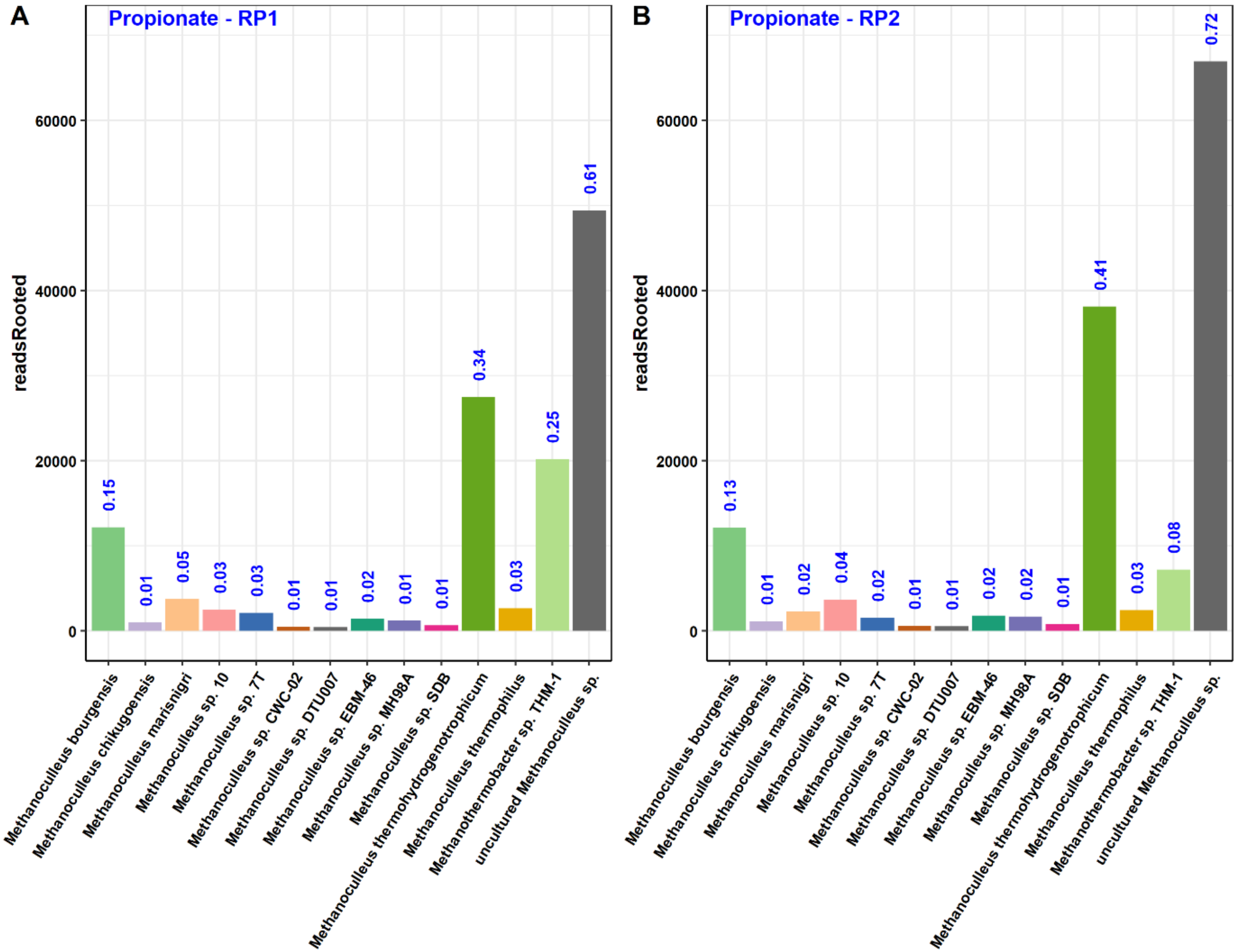

Figure S16. Metagenomics classification of samples from reactors RP1 and RP2 using kraken2 against a custom database for the genera *Methanoculleus* and *Methanothermobacter*.

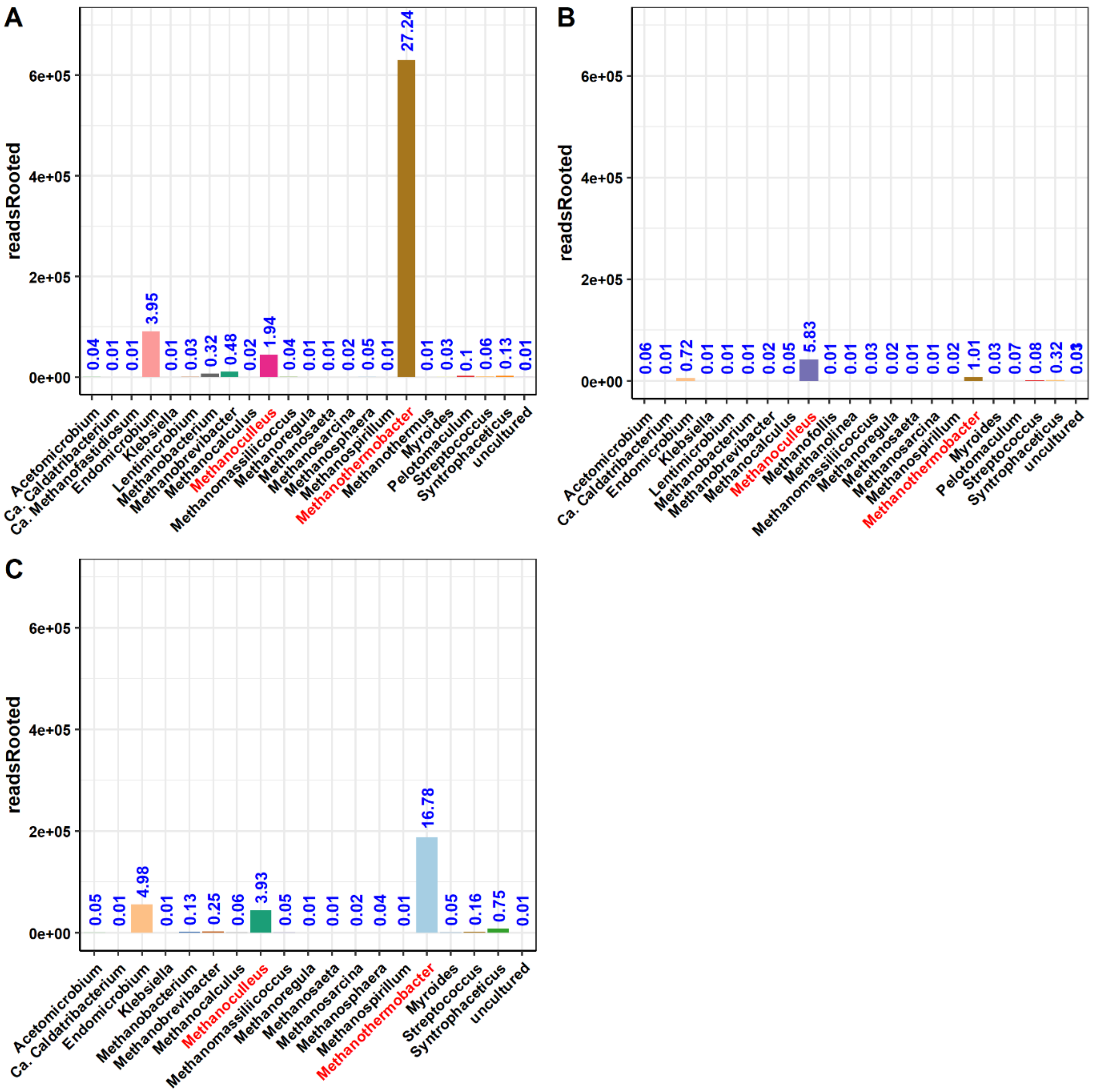

Figure S17. Comparison of transcript abundance in metatranscriptomics samples A) B01, B) B03 and C) B09 for methanogens *Methanoculleus* and *Methanothermobacter* using the default kraken2 database.

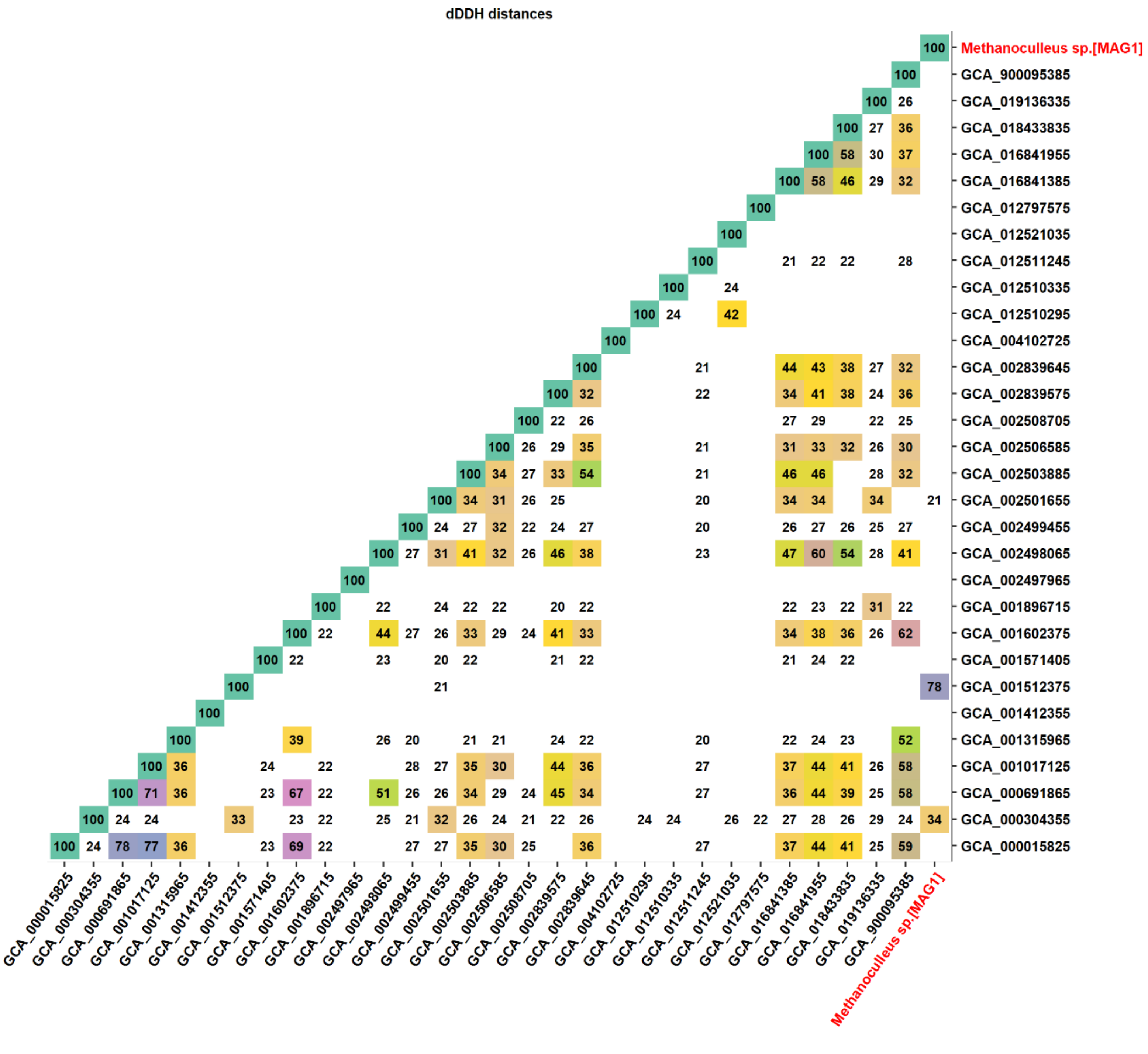

Figure S18. Comparison of genome similarities of **MAG1** with genomic assemblies belonging to the genus *Methanoculleus* and closely related species based on digital DNA-DNA hybridisation (dDDH).

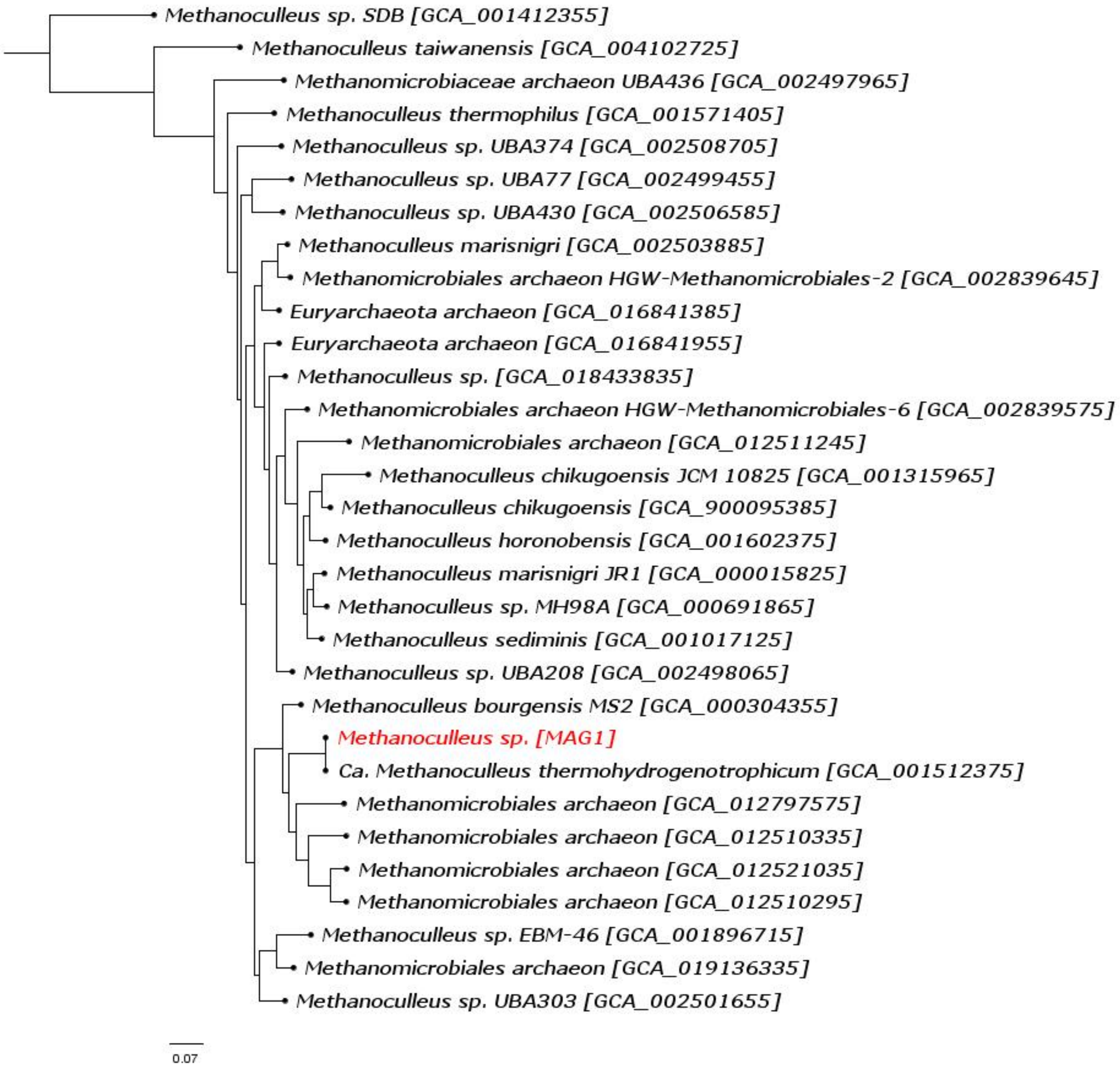

Figure S19. Species tree based on orthologous sequences of **MAG1** and other species and candidates in the genus *Methanoculleus* and closely related species.

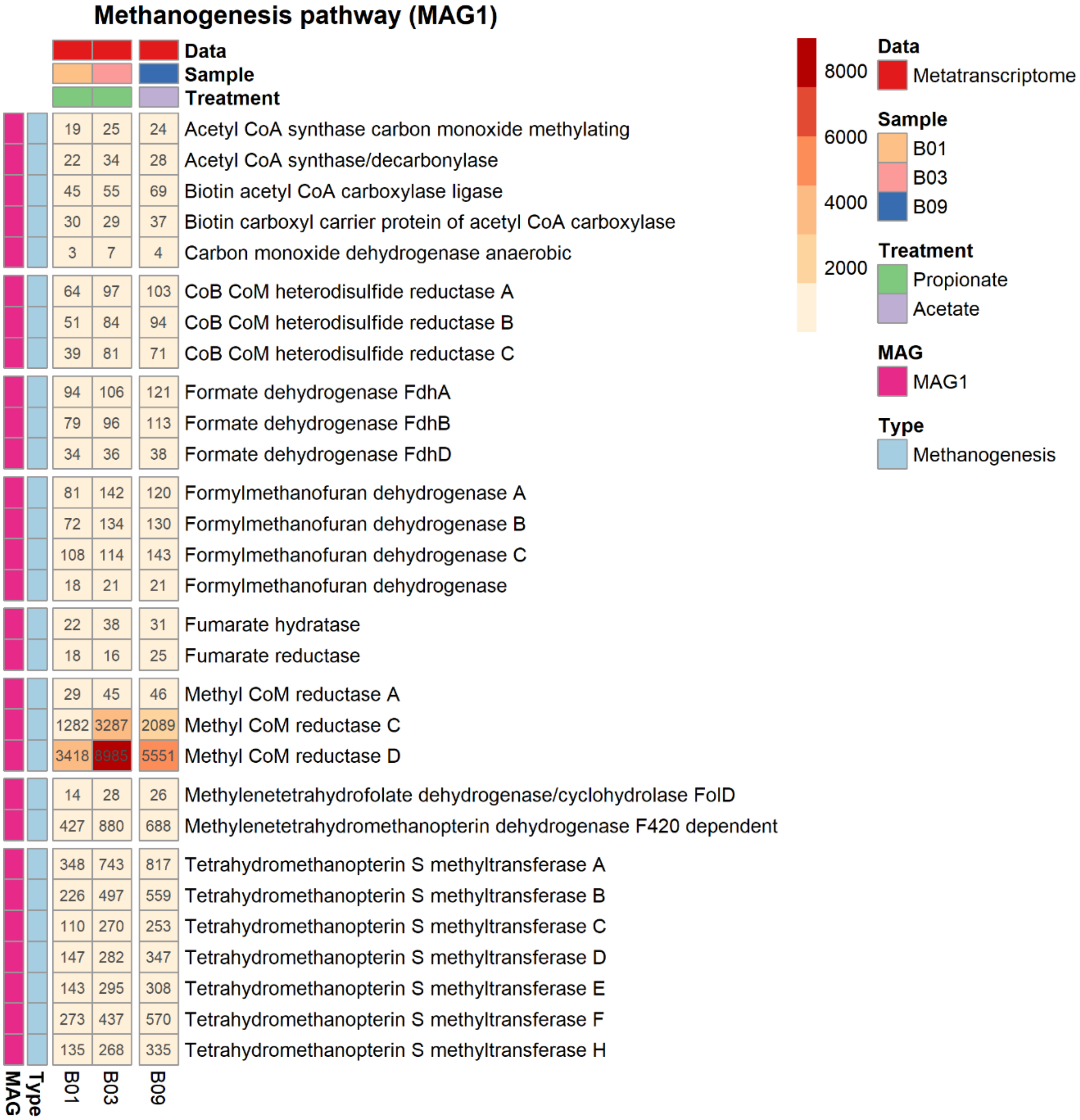

Figure S20. Metatranscriptomics expression profile of the hydrogenotrophic pathway (based on transcripts per million (TPM) counts) for the metagenomic assembled genome (MAG) of the methanogen **MAG1** in propionate (B01 - faster propionate degradation, B03 - slower propionate degradation) versus acetate batch assay (B09).

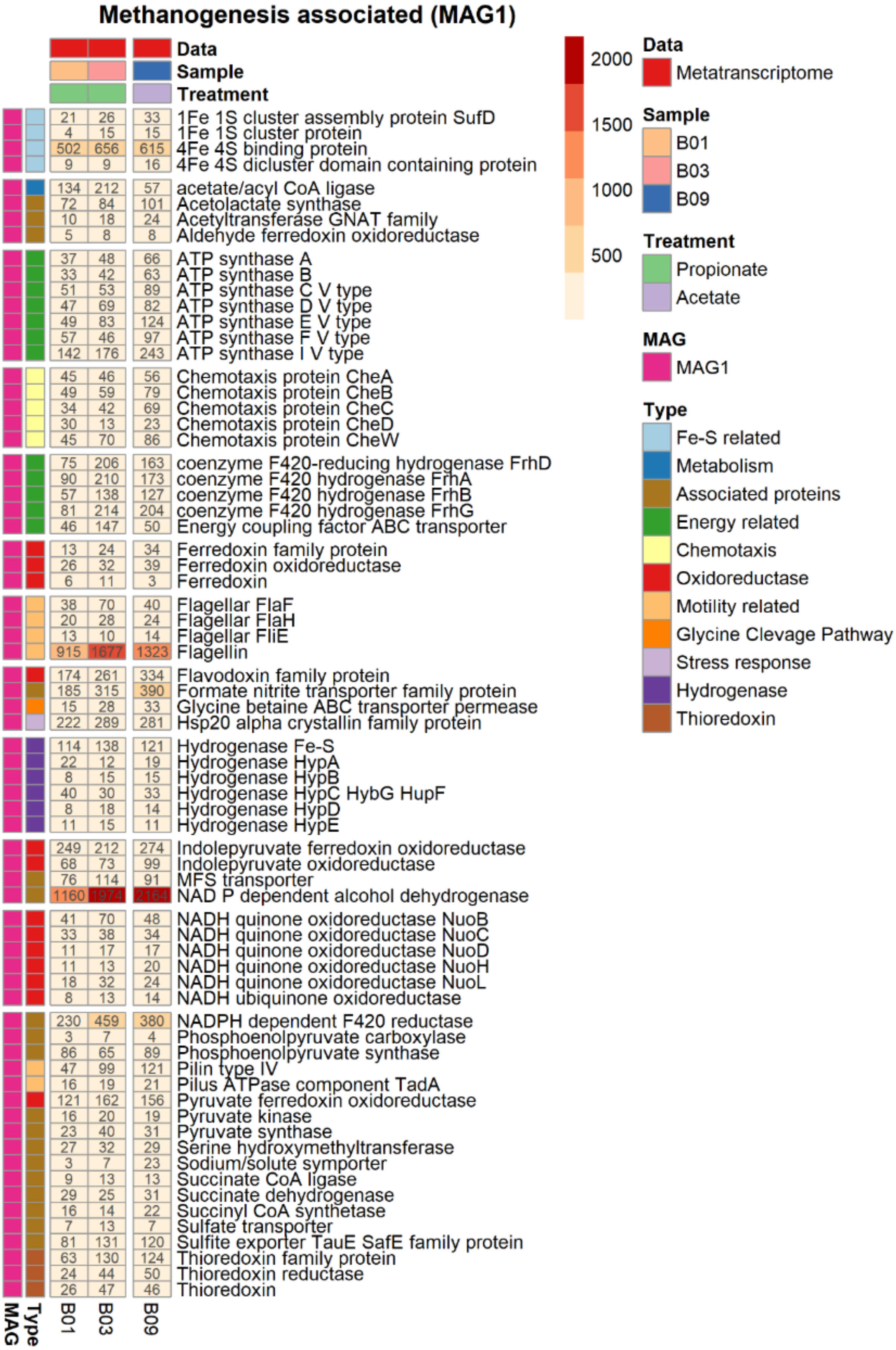

Figure S21. Metatranscriptomics expression profile of hydrogenotrophic pathway-associated proteins (based on transcripts per million (TPM) counts) for the metagenomic assembled genome (MAG) of the methanogen **MAG1** in propionate (B01 - faster propionate degradation, B03 - slower propionate degradation) versus acetate batch assay (B09).

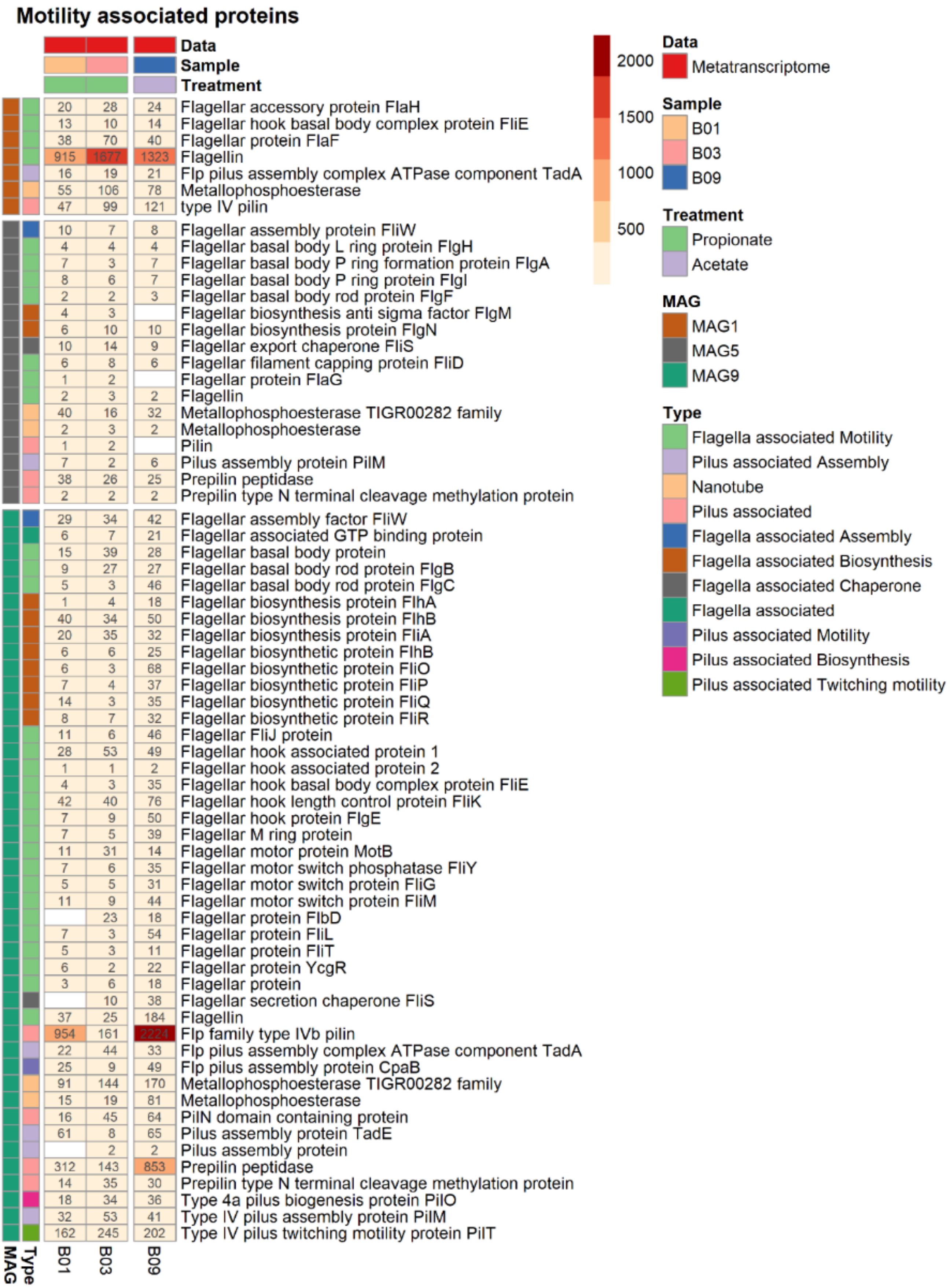

Figure S22. Metatranscriptomics expression profile of motility-associated proteins (based on transcripts per million (TPM) counts) in propionate (B01 - faster propionate degradation, B03 - slower propionate degradation) versus acetate batch assay (B09) for the metagenomic assembled genomes (MAGs).

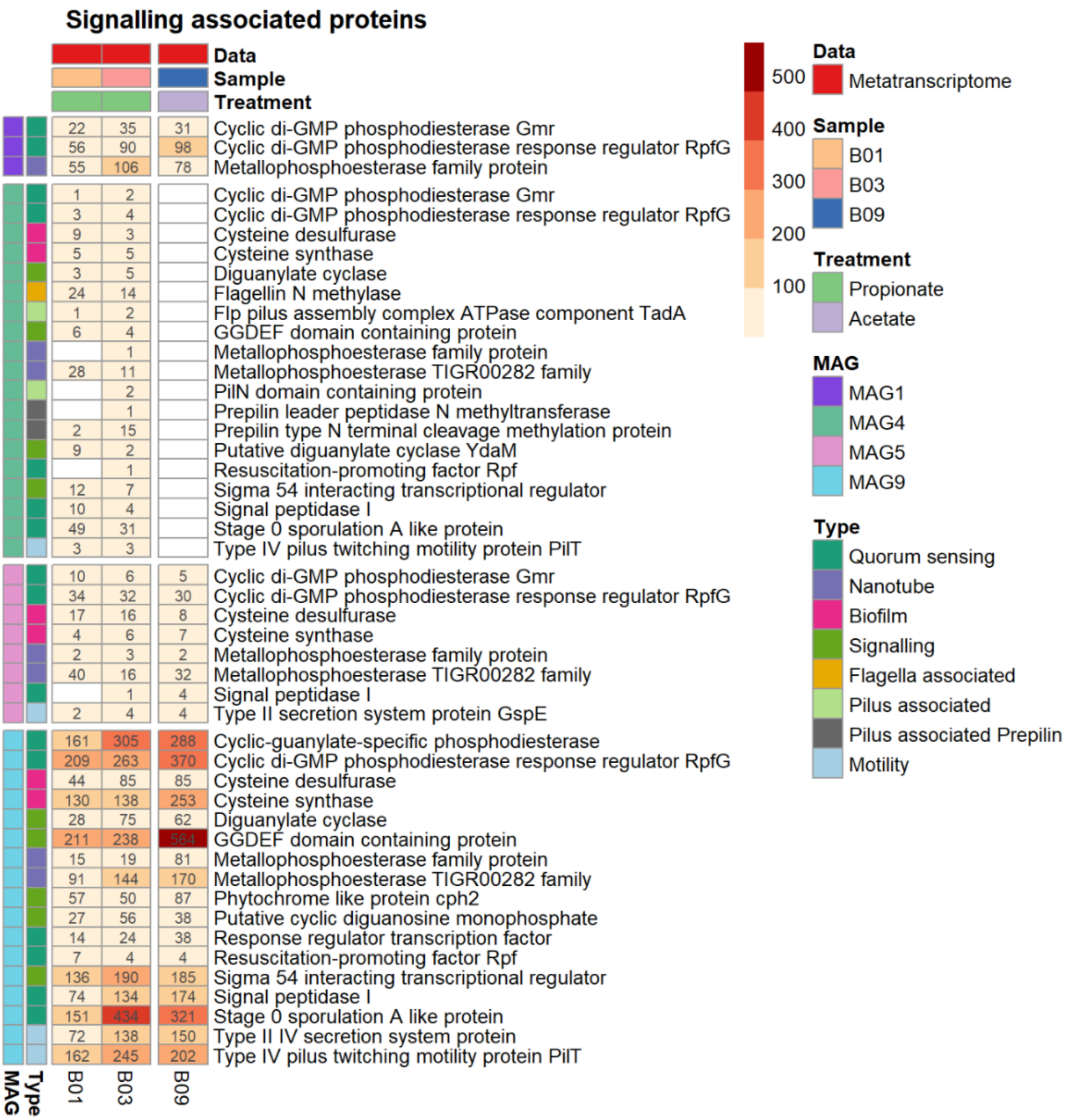

Figure S23. Metatranscriptomics expression profile of signalling- and cellular communication-associated proteins (based on transcripts per million (TPM) counts) in propionate (B01 - faster propionate degradation, B03 - slower propionate degradation) versus acetate batch assay (B09) for the metagenomic assembled genomes (MAGs).

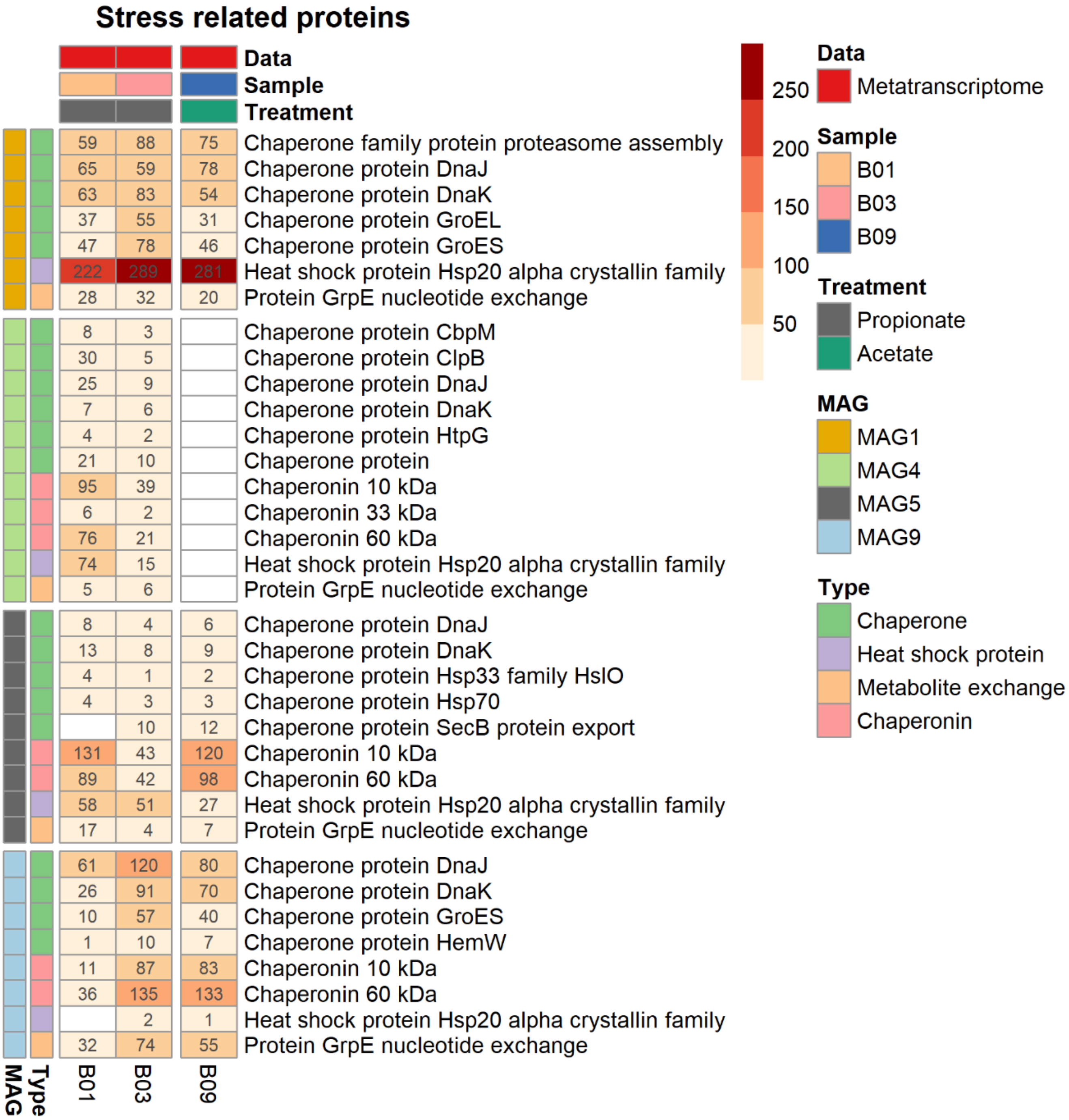

Figure S24. Metatranscriptomics expression profile of stress-related proteins (based on transcripts per million (TPM) counts) in propionate (B01 - faster propionate degradation, B03 - slower propionate degradation) versus acetate batch assay (B09) for the metagenomic assembled genomes (MAGs).
