# Supplementary material for: Syntrophic entanglements for propionate and acetate oxidation under thermophilic and high-ammonia conditions
Source: ISME J. 2023 Sep 7;17(11):1966–78. doi: 10.1038/s41396-023-01504-y (PMC10579422; doi:10.1038/s41396-023-01504-y)
Supplement: Supplementary file 3 — Supplementary tables [file 41396_2023_1504_MOESM3_ESM.docx]

**Table S1.** Operating conditions for the large-scale biogas digesters that were the inoculum source for the thermophilic (52°C) chemostats in the present study

| Trial | Thermophilic |
| --- | --- |
| Digester affiliation | Kungsängens gård |
| Digester type | Continuously stirred tank reactor (CSTR) |
| Substrate | Industrial food waste, animal manure |
| Temperature (°C) | 52 |
| Organic loading rate (OLR, g VS L^-1^day^-1^) | 3.5 |
| Hydraulic retention time (HRT, days) | 30 |
| NH_4_^+^-N (g L^-1^) | 3.2 |
| NH_3_ (g L^-1^) | 0.6 |
| Trace element supplementation | 1.5 L Kemira BDP-866^1^ m^-3^ substrate |
| Volatile fatty acid (VFA) (g L^-1^) | 3.8 |

^1^Commercial product containing iron (Fe^2+^/Fe^3+^), cobalt, nickel, selenium, tungsten and hydrochloric acid.

**Table S2.** Process data for parallel propionate-fed (RP1, RP2) and acetate-fed (RA1, RA2) reactors. Average values for 21-33 data points during stable operation, *i.e.*, between days 50 and 450

| Reactor | Substrate  (M) | Conc.  (g L^-1^) | Propionate in outflow  (g L^-1^) | Acetate in outflow  (g L^-1^) | Gas production rate  (NmL d^-1^) | Methane content (%) | H_2_S  (ppm) | pH |
| --- | --- | --- | --- | --- | --- | --- | --- | --- |
| RP1 | Sodium propionate (0.1) | Propionate (9.6) | 1.0 ± 0.1 | 0.8 ± 0.1 | 91±1 | 70 ± 2 | 1394 ± 84 | 8.1 ± 0.03 |
| RP2 |  |  | 1.4 ± 0.2 | 1.2 ± 0.1 | 69±1 | 66 ± 2 | 1928 ± 106 | 8.1 ± 0.03 |
| RA1 | Sodium acetate (0.1) | Acetate (8.9) | na | 0.9 ± 0.1 | 68±1 | 66 ± 2 | 526 ± 106 | 8.3 ± 0.03 |
| RA2 |  |  | na | 0.7 ± 0.1 | 51±1 | 62 ± 2 | 462 ± 102 | 8.3 ± 0.03 |

na = not applicable

**Table S3.** Calculation of the stoichiometry of formed methane and acid consumption in the propionate-fed batches B01-B02 and B03-B04 at day 46 and 86, respectively

| Attribute | Units | Batches | |
| --- | --- | --- | --- |
|  |  | B01/B02 | B03/B04 |
| n_propionate_IN_ Propionate added | mmol | 15.6 | 14.9 |
| n_acetate_IN_ Acetate originating from the inoculum medium | mmol | 10.5 | 8.9 |
| n_propionate_OUT_ Propionate remaining at the day of analysis | mmol | 0 | 2.5 |
| n_acetate_OUT_ Acetate remaining at the day of analysis | mmol | 0 | 3.0 |
| Expected methane yield^1^ | mmol | 37.8 | 27.6 |
| Methane produced | mmol | 37.8 | 29.6 |

^1^The expected methane yield was calculated according to:

n_CH4_ = (n_propionate_IN_ ⋅ 7/4) + (n_acetate_IN_ ⋅ 1/1) - (n_propionate_OUT_ ⋅ 7/4) - (n_acetate_OUT_ ⋅ 1/1)

where the n_propionate_ and n_acetate_ represent the number of mol of the acids added or consumed. The balanced equations are as follow:

**Propionate oxidation**

CH_3_CH_2_COO^-^ + 3H_2_O + H^+^ $\to$ CH_3_COO^-^ + HCO_3_^-^ + 3H_2_ + 2H^+^

**Acetate oxidation**

CH_3_COO^-^ + 4H_2_O + H^+^ $\to$ 2HCO_3_^-^ + 4H_2_ + 2H^+^

**Sum**

CH_3_CH_2_COO^-^ + 7H_2_O + H^+^$\to$ 3HCO_3_^-^ + 7H_2_ + 3H^+^ (eq. I)

**Methane formation**

4H_2_ + HCO_3_^-^ + H^+^$\to$ CH_4_ + 3H_2_O (eq. II)

Multiplying eq. I by 4 and eq. II by 7 to equalise the two reactions gives:

4CH_3_CH_2_COO^-^ + 28H_2_O + 4H+ $\to$ 12HCO_3_^-^ + 28H_2_ + 12H^+^

28H_2_ + 7HCO_3_^-^ + 7H^+^$\to$ 7CH_4_ + 21H_2_O

**Sum**

4CH_3_CH_2_COO^-^ + 7H_2_O + 4H+$\to$ 7CH_4_ + 5HCO_3_^-^ + 7H_2_ + 5H^+^ (eq. III)

**Table S4.** Comparison of average values for enrichment reactors in mesophilic and thermophilic conditions

|  | Mesophilic reactors  (Singh *et al*., 2021) | Thermophilic reactors  (this study) |
| --- | --- | --- |
| Temperature (°C) | 37 | 52 |
|  | 7.9-8.1 | 8.1-8.2 |
| Acetate (g L^-1^) | 0.5 | 0.8 |
| Propionate (g L^-1^) | 0.6 | 1.2 |
| Methane content (%) | 35-78 | 56-72 |
| Ammonia (g L^-1^) | 0.4-0.6 | 0.7-0.9 |

**Table S5.** Average rate of propionate and acetate degradation in duplicate batch assays originating from propionate-fed (RP1, RP2) and acetate-fed (RA1, RA2) reactors

| Batch | Origin of culture | Acid fed to the batch | Hydrogen partial pressure (Pa) | Rate of degradation, g L^-1^ day  (measured range) | |
| --- | --- | --- | --- | --- | --- |
|  |  |  |  | Propionate | Acetate |
| B01-B02 | RP1 | Propionate | 4-12 (peak at 30 at one time point) | 0.16 (day 0-11) | 0.10 (day 18-39) |
| B03-B04 | RP2 | Propionate | 3.5-4.0 | 0.02 (day 0-112) | 0.05 (day 4-18)^1^ |
| B05-B06 | RP1 | Acetate | 6-32 | - | 0.10 (day 4-25) |
| B07-B08 | RP2 | Acetate | 5-20 | - | 0.12 (day 4-25) |
| B09-B10 | RA1 | Acetate | 8-24 | - | 0.08 (day 11-39) |
| B11-B12 | RA2 | Acetate | 9-30 | - | 0.13 (day 0-18) |

^1^A feasible reason for the lower rate is lower rate of acetate formation and less accumulated acetate in batches B03-04 than in B01-03.

**Table S6.** Thermodynamic parameters and Gibbs free energy (ΔG) of the three reactions involved in syntrophic conversion of propionate into methane under high-ammonia conditions

| Reaction | ΔH^0^ (kJ) | ΔG^0^ (kJ) | ΔG^0^_52_ (kJ) |
| --- | --- | --- | --- |
| Propionate degradation (CH_3_CH_2_COO^-^ + 3H_2_O → CH_3_COO^-^ + 3H_2_ + HCO_3_^-^ + H^+^) | 205.1 | 73.7 | 61.8 |
| Acetate oxidation (CH_3_COO^-^ + 4H_2_O → 4H_2_ + 2HCO_3_^-^ + H^+^) | 270.7 | 54.9 | 35.4 |
| Hydrogenotrophic methanogenesis (4H_2_ + HCO_3_^-^ + H^+^ → CH_4_ + 3H_2_O | -252.9 | -130.8 | -119.7 |

**Table S7.** Taxonomic classification of selected metagenome assembled genomes (MAGs) using GTDB-tk and their attributes

| Attributes | MAG1 | MAG4 | MAG5 | MAG9 |
| --- | --- | --- | --- | --- |
| Kingdom | Archaea | Bacteria | Bacteria | Bacteria |
| Phylum | Halobacteriota | Firmicutes_B | Synergistota | Firmicutes_B |
| Class | Methanomicrobia | Desulfotomaculia | Synergistia | Syntrophomonadia |
| Order | Methanomicrobiales | Desulfotomaculales | Synergistales | Thermacetogeniales |
| Family | Methanoculleaceae | Pelotomaculaceae | Acetomicrobiaceae | Thermacetogeniaceae |
| Genus | Methanoculleus | DTU098^1^ | Acetomicrobium | DTU068^1^ |
| Species | Methanoculleus thermohydrogenotrophicum | DTU098 sp001512635^1^ | NA | NA |
| Length (bp) | 2,620,954 | 3,229,521 | 2,199,980 | 2,964,479 |
| Contigs (count) | 10 | 6 | 16 | 14 |
| Completeness (%) | 99.02 | 97.47 | 100 | 100 |
| Contamination (%) | 0 | 2.43 | 0 | 15.49 |
| Strain heterogeneity (%) | 0 | 12.50 | 0 | 89.74 |
| GC content (%) | 58.73 | 49.5 | 44.9 | 45.1 |
| Predicted genes (count) | 2,702 | 3,126 | 2,383 | 3,069 |
| Annotated genes (count) | 1,762 | 2,150 | 1,687 | 2,284 |
| Hypothetical proteins (count) | 940 | 976 | 696 | 785 |
| Coding density (%) | 81.76 | 83.00 | 88.40 | 85.70 |
| 16S rRNA (count) | 3 | 3 | 2 | 3 |
| tRNA (count) | 45 | 56 | 54 | 57 |

^1^indicate the taxonomic rank as temporary place holder taxon name, which is not validated or validly published

**Table S8.** Number of 16S rRNA gene copies of Methanomicrobiales and Methanobacteriales in the continuously-fed reactors (RP1, RP2, RA1, RA2) and in the batch assays, determined using quantitative polymerase chain reaction (qPCR) analyses

|  | Methanomicrobiales  (gene copies L^-1^)^1^ | Methanobacteriales  (gene copies L^-1^) |
| --- | --- | --- |
| RP1 | 10^7^-10^8^ → 10^5^ | 10^5^-10^7^ |
| RP2 | 10^7^-10^8^ → 10^5^ | 10^5^-10^7^ |
| RA1 | 10^7^-10^8^ → 10^5^ | 10^5^-10^7^ |
| RA2 | 10^7^-10^8^ → 10^5^ | 10^5^-10^7^ |
| B01-B02 | 6^.^10^6^ ± 1^.^10^3^ | 2^.^10^6^ ± 2^.^10^2^ |
| B03-B04 | 6^.^10^6^ ± 9^.^10^2^ | 5^.^10^4^ ± 7^.^10^1^ |
| B05-B06 | 6^.^10^6^ ± 1^.^10^1^ | 2^.^10^6^ ± 1^.^10^1^ |
| B07-B08 | 8^.^10^6^ ± 1^.^10^1^ | 9^.^10^4^ ± 7^.^10^2^ |
| B09-B10 | 2^.^10^6^ ± 9^.^10^2^ | 6^.^10^5^ ± 1^.^10^3^ |
| B11-B12 | 3^.^10^6^ ± 9^.^10^2^ | 5^.^10^5^ ± 9^.^10^2^ |

^1^Arrow indicates changes over time in the continuously-fed reactors.
